# A Transcriptomic Atlas of the Ectomycorrhizal Fungus *Laccaria bicolor*

**DOI:** 10.3390/microorganisms9122612

**Published:** 2021-12-17

**Authors:** Joske Ruytinx, Shingo Miyauchi, Sebastian Hartmann-Wittulsky, Maíra de Freitas Pereira, Frédéric Guinet, Jean-Louis Churin, Carine Put, François Le Tacon, Claire Veneault-Fourrey, Francis Martin, Annegret Kohler

**Affiliations:** 1 Institut National de Recherche pour d’Agriculture, d’Alimentation et l’Environnement (INRAE), UMR1136 Interactions Arbres/Microorganismes, INRAE Grand Est-Nancy, Université de Lorraine, 54280 Champenoux, France; smiyauchi@mpipz.mpg.de (S.M.); sebastian.wittulsky@bayer.com (S.H.-W.); mairadefreitaspereira@gmail.com (M.d.F.P.); frederic.guinet@univ-lorraine.fr (F.G.); churin.jean-louis@orange.fr (J.-L.C.); francois.letacon@gmail.com (F.L.T.); claire.veneault-fourrey@inrae.fr (C.V.-F.); annegret.kohler@inrae.fr (A.K.); 2 Research Groups Microbiology and Plant Genetics, Department of Bioengineering Sciences, Vrije Universiteit Brussel, 1050 Brussel, Belgium; 3 Department of Plant Microbe Interactions, Max Planck Institute for Plant Breeding Research, 50829 Cologne, Germany; 4 Institute for Plant Sciences, University of Cologne, 50829 Cologne, Germany; 5 Centre for Environmental Sciences, Environmental Biology, Hasselt University, 3590 Diepenbeek, Belgium; carine.put@uhasselt.be

**Keywords:** ectomycorrhiza, symbiosis, carpophore, nitrogen, phosphorus, transcriptome

## Abstract

Trees are able to colonize, establish and survive in a wide range of soils through associations with ectomycorrhizal (EcM) fungi. Proper functioning of EcM fungi implies the differentiation of structures within the fungal colony. A symbiotic structure is dedicated to nutrient exchange and the extramatricular mycelium explores soil for nutrients. Eventually, basidiocarps develop to assure last stages of sexual reproduction. The aim of this study is to understand how an EcM fungus uses its gene set to support functional differentiation and development of specialized morphological structures. We examined the transcriptomes of *Laccaria bicolor* under a series of experimental setups, including the growth with *Populus tremula x alba* at different developmental stages, basidiocarps and free-living mycelium, under various conditions of N, P and C supply. In particular, N supply induced global transcriptional changes, whereas responses to P supply seemed to be independent from it. Symbiosis development with poplar is characterized by transcriptional waves. Basidiocarp development shares transcriptional signatures with other basidiomycetes. Overlaps in transcriptional responses of *L. bicolor* hyphae to a host plant and N/C supply next to co-regulation of genes in basidiocarps and mature mycorrhiza were detected. Few genes are induced in a single condition only, but functional and morphological differentiation rather involves fine tuning of larger gene sets. Overall, this transcriptomic atlas builds a reference to study the function and stability of EcM symbiosis in distinct conditions using *L. bicolor* as a model and indicates both similarities and differences with other ectomycorrhizal fungi, allowing researchers to distinguish conserved processes such as basidiocarp development from nutrient homeostasis.

## 1. Introduction

Forests are dominant terrestrial ecosystems covering 34.31% of land area globally (Copernicus, Global Land Cover viewer) and provide services ranging from timber production to climate regulation and recreation [1]. Tree-associated fungal communities are important determinants of forest productivity in terms of resilience, atmospheric carbon fixation and retention [2]. Forests house multiple fungi of different ecological guilds, including pathogens, organic matter decomposers and ectomycorrhizal (EcM) species [2]. In particular, EcM fungi have been shown to be pervasive in determining plant co-existence, dispersal and seedling establishment [3,4]. EcM fungi evolved several times independently from saprotrophs and wood decayers [5,6] and are mutualists. Their hyphae surround tree root tips with a mantle, penetrate in between cortex cells and extend into the soil to collect water and essential nutrients in exchange for photosynthesis-derived sugar [7]. Besides, EcM fungi provide additional benefits to their host plant, such as protection from biotic and abiotic stressors [8].

The accumulation of sequenced genomes of EcM species has led to discoveries of particular genomic features and gene sets separating them from their ancestors with a saprotrophic or pathogenic lifestyle [6,9]. Typical genomes of EcM fungi contain (i) a reduced set of plant cell wall-degrading enzyme-encoding genes compared to their saprotrophic ancestors and (ii) lineage-specific small secreted protein (SSP)-encoding genes [6,10,11,12]. Across EcM fungal species, the expression of the lineage specific SSPs, so-called MiSSPs (Mycorrhiza-induced Small Secreted Proteins) is induced in EcM root tips. A handful of MiSSPs were functionally characterized and were shown to interact with the host plant’s gene regulation and stress responses as effector molecules [13,14,15]. For others, such as LbMiSSP8, their contribution at the plant–fungal interface is less clear and their role in EcM functioning might not be restricted to EcM establishment but might extend towards other morphological structures or the regulation of environmental responses [16]. The evolutionarily retained carbohydrate active enzyme (CAZyme)-encoding gene set, is most likely no longer used by EcM fungi to degrade soil organic matter or plant material as a carbon source but rather deployed to access nitrogen and/or modify plant and fungal cell walls during morphogenesis of symbiotic EcM roots [17,18,19]. A new family of lytic polysaccharide mono-oxygenases, which are copper-containing oxygenases acting on cell wall polysaccharides such as cellulose and chitin, was discovered in fungi and named X325. Surprisingly, members of this family, including *L. bicolor* X325 which is expressed in ectomycorrhizal root-tips, no longer have the ability to degrade polysaccharides but most likely adopted a new function in Cu transport [20].

Indeed, EcM function is not restricted to the morphogenesis of a symbiotic organ but involves differentiation within the fungal colony to support nutrient transport in response to environmental conditions, as well as fungal and host plant’s needs. Biochemical and molecular responses allow EcM fungi to restore homeostatic equilibrium upon fluctuations in nutrient availability within particular concentration ranges. Any environmental change beyond that homeostatic concentration range results in visible effects such as reductions in growth and symbiotic function. In addition, differentiation into fruiting bodies to assure the final stages of sexual reproduction and dispersal of meiospores is an essential part of EcM functioning. In this study, we aim at deciphering the transcription patterns across developmental stages and nutritional conditions in order to obtain novel insights into the gene regulation of EcM fungi. The mechanisms that EcM species use to regulate their gene set to support morphological and functional differentiation within a colony and to respond to a host plant or environmental change are largely unknown. This information is required to gain a better understanding of their mutual beneficial lifestyle. 

## 2. Material and Methods

### 2.1. Fungal Isolate and Maintenance of Cultures

*Laccaria bicolor* S238N (Maire) P.D. Orton was used in all experiments. A culture of the isolate was maintained on modified Pachlewski agar medium P5 [21] at 25 °C in the dark with biweekly subculturing.

### 2.2. Growth in Different Nutritional Conditions

*L. bicolor* mycelium agar plugs (0.5 cm^2^) were inoculated on a cellophane membrane covering modified MMN medium (Modified Melin Norkrans containing 0.45 mM CaCl_2_, 0.43 mM NaCl, 3.67 mM KH_2_PO_4_, 1.89 mM (NH_4_)_2_HPO_4_, 0.61 mM MgSO_4_·7H_2_O, 0.037 µM FeCl_3_·6H_2_O, 0.003 µM thiamine HCl, 0.5 mM KCl, 0.25 mM H_3_BO_3_, 50 µM MnSO_4_·7H_2_O, 20 µM ZnSO_4_·7H_2_O, 5 µM CuSO_4_·5H_2_O, 0.146 µM (NH_4_)_6_Mo_7_O_24_·4H_2_O, 0.5% glucose, 0.5% malt extract, 1% casein hydrolysate, 2% agar and 0.1% MES, according to [22]) and pre-cultured for two weeks in the dark at 25 °C. After pre-culture, fungal colonies were transferred to new plates containing control modified MMN medium or modified MMN medium adjusted for N (−N = all NH_4_ salts removed and KH_2_PO_4_ supplemented to correct final PO_4_ concentration; +N = supplemented with 25 mM NH_4_Cl_2_), P (−P = all PO_4_ salts removed and NH_4_Cl_2_ supplemented to correct final NH_4_ concentration; +P = supplemented with 25 mM KH_2_PO_4_) or C (−C = glucose reduced to 0.02%; +C = glucose supplemented to 2%) content to induce nutrient deficiency or surplus. Plates were incubated in the dark for an additional week at 25 °C. Subsequently, pictures were taken and the mycelium was snap frozen in liquid nitrogen. The samples were stored at -80 °C until further RNA extraction or lyophilization.

### 2.3. Plant Material and Fungal–Plant Co-Cultures

*Laccaria bicolor* S238N and *Populus tremula x alba* 717-1B4 in vitro co-cultures were established according to the sandwich system described by [23], with small modifications. In brief, *L. bicolor* was pre-cultured in the dark at 25 °C for one week on sugar-reduced Pachlewski agar medium P20. *P. tremula x alba* micro-cuttings were stimulated to root for 1 week on solid Murashige and Skoog (MS) medium supplemented with 2 mg/L of IBA (Indol-3-butyric acid) and subsequently transferred to cellophane-covered MS plates. After three weeks of incubation at 25 °C with a 16 h photoperiod, poplar explants were transferred to cellophane-covered P20 medium supplemented with 0.1% MES. To allow plant–fungal interaction to develop, the cellophane covered with *L. bicolor* pre-cultures was placed in an inverted position on the roots. Control plates, containing no plants (i.e., free-living mycelium (FLM)), were prepared and handled the same way as co-cultures. All plates were incubated at 20 °C with a 16 h photoperiod. The samples (100 mg fresh weight) for RNA extraction were taken in triplicate at 0, 3, 7, 14 and 21 days of (co-)culture from FLM, EcM (i.e., all fungal and mixed plant–fungal material within 0.5 cm distance of a root) and extramatrical mycelium (i.e., all fungal material at a distance of >0.5 cm of a root), snap frozen in liquid nitrogen and stored at −80 °C. Root tips were sampled for microscopy at the same time points and fixated overnight at 4 °C in 4% paraformaldehyde. 

### 2.4. Basidiocarp Production and Sampling

*L. bicolor* basidiocarps were harvested from a nursery experiment of inoculated Douglas fir seedlings as described by [24]. Cap and stipe were separated from early-, medium- and late-stage mushrooms, immediately frozen in liquid nitrogen and stored at −80 °C.

### 2.5. P Determinations

Lyophilized mycelium (±40 mg DW) was acid-digested at 110 °C using 3x HNO_3_ and HCl subsequently. Inductively coupled plasma optical emission spectroscopy (ICP-OES) was used to determine P concentrations of the samples in a 2% HCl solution. P determinations were performed for at least three independent replicates per experimental condition. Mean values were calculated and a one-way ANOVA, followed by Holm–Sidak multiple comparisons versus the control, was run to assess differences in P accumulation in response to P availability in the growth medium.

### 2.6. Microscopy

Overnight, paraformaldehyde-treated root tips were washed for 3 × 5 min in phosphate buffered saline (PBS) and embedded in 4% agarose prior to 25 µm transversal sectioning with a vibratome (Leica (Wetzlar, Germany), VT1000S). Sections at a distance of 150–250 µm from the root tip were collected, exposed to a droplet of 10 µM WGA-Alexa 488 for 1 h to selectively stain the fungal mycelium, washed 3x in PBS and stained with a droplet of 10 µM propidium iodide (PI, 5 min) followed by an additional PBS wash step. Stained root sections were mounted on a microscope slide. A confocal microscope (Zeiss (Jena, Germany), LSM780—Axio Observer) was used for the viewing of five independent replicates per experimental condition. Afterwards, 514 nm and 488 nm lasers were used for excitation; a 589–695 BP filter and a 508–535 BP filter were used for detection of PI and Alexa 488, respectively. The images were recorded and processed using ZEN 2 lite software (Zeiss).

### 2.7. RNA Extractions and RNAseq

Total RNA was extracted from fungal and mixed plant–fungal biomass (100 mg) using the RNeasy Plant Mini Kit (Qiagen, Hilden, Germany). Biomass was ground in liquid nitrogen prior to extraction and an on-column DNAse I treatment was included in the protocol. For samples containing poplar roots (EcM), extraction buffer RLC was supplemented with 2% PEG (polyethylene glycol) 8000. Total RNA of basidiocarp stipes and caps was extracted using a CTAB (cetyltrimethylammonium bromide)-based protocol described in [25], including a LiCl precipitation of total RNA. RNA concentration of all samples was determined spectrophotometrically and integrity was assessed using the Biorad Experion and RNA StdSens kit and chips. Three replicates were used for RNA-seq sequencing. The preparation of libraries from total RNA (TruSeq Stranded mRNA Kit Illumina), 2 × 100 bp Illumina HiSeq2000 sequencing for the basidiocarp samples and 2 × 150 bp Illumina HiSeq3000 sequencing for all other samples were performed on the GET platform (Génopole Toulouse Midi-Pyrénées, Auzeville, France) following their standard protocol.

Raw reads were trimmed for low quality (quality score, 0.05), Illumina adapters and sequences shorter than 15 nucleotides and aligned to the *L. bicolor* v2 reference transcripts available in the JGI database https://mycocosm.jgi.doe.gov/Lacbi2/Lacbi2.home.html (last accessed on 6 December 2021) using CLC Genomics Workbench v8. For read mapping, the CLC genomic workbench parameters were the following: minimum length fraction, 0.9; minimum similarity fraction, 0.8; mismatch cost = 2; insertion cost = 3; deletion cost = 3; and the maximum number of hits for a read was set to 10. The unique and total mapped read numbers for each transcript were determined and then normalized to RPKM (reads per kilobase of exon model per million mapped reads). We calculated the differential expression of genes with DESeq2 [26]. We used SHIN+GO to determine co-regulated genes for the three individual experiments, including (1) growth in different nutritional conditions, (2) kinetics of plant–fungal co-culture and (3) basidiocarp development [9,27,28,29] (Supplementary Information). To identify similarities and dissimilarities in expression patterns over all tested conditions, normalized read counts were used as an entry for the web-based visualization and analysis tool Morpheus (https://software.broadinstitute.org/morpheus; last accessed on 21 October 2021). Hierarchical clustering was run using default settings and the data were visualized in a color-coded heatmap. TopGO [30] was used to conduct the enrichment analyses for Gene Ontology. A webtool (http://bioinformatics.psb.ugent.be/webtools/Venn/; last accessed on 21 October 2021) was used for the generation of Venn diagrams.

## 3. Results

### 3.1. Effect of Nutrition on Growth and Mycelial P Accumulation

**Figure 1:**
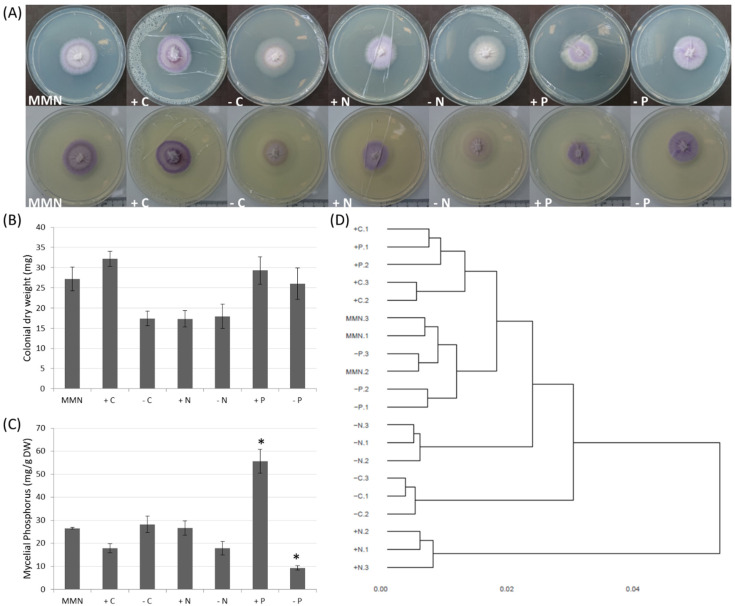
Responses of *L. bicolor* to different nutritional conditions. (**A**) On MMN medium pre-cultured mycelia grown in 9 cm diameter petri dishes for one week on control MMN medium or MMN medium adjusted for glucose (±C), ammonium (±N) or phosphate (±P) to induce nutrient deficiency or surplus. Upper and lower panel represent the same plates on a different background. (**B**) Mean dry weight ± S.E. of three colonies. (**C**) Average phosphorus accumulation ± S.E. for five colonies. Values marked by * differ significantly from values measured in MMN control medium (one-way ANOVA; Holm–Sidak multiple comparisons versus control; *p* < 0.01). (**D**) Hierarchical clusters of overall transcriptomic responses of 20 biological replicates grown in 7 different nutritional conditions.

Variations in the composition of the culture medium, i.e., reduced or increased glucose and NH_4_^+^ or PO_4_^−^ concentration, did not result in any significant effect on the growth of *L. bicolor*. The size of the colonies and colonial dry weight were not significantly different, despite having grown with different nutritional conditions (Figure 1A,B). Nonetheless, mycelia grown on medium adjusted for NH_4_^+^ (+N/−N) or with reduced glucose (−C) tended to have lower biomass. Effects on metabolism were clearly visible as changes in colonial pigmentation. Color shades from white to dark violet marked the colonies depending on the composition of the growth medium (Figure 1A). *L. bicolor* colonies grown on control MMN medium were violet with a small peripheral ring of white mycelium at the edge of the colony. Variations in glucose, NH_4_^+^ and PO_4_^−^ concentration resulted in the local absence of the violet pigment in mycelium grown after the transfer of the colony from control MMN to a different nutritional condition (violet center surrounded by a broad white ring as for reduced glucose (−C) and surplus PO_4_^−^ (+P); Figure 1A) or in the absence of the pigment with a de-coloration of the MMN pre-cultured mycelium (whole white colony, as for reduced NH_4_^+^ (−N); Figure 1A). The accumulation of P within the mycelium changed according to PO_4_^−^ concentration in the growth medium, with mycelia grown in PO_4_^−^ surplus showing the highest P accumulation (Figure 1C). 

### 3.2. Nutrition-Regulated Transcriptome

Transcripts of 14,733 out of 23,130 *L. bicolor* gene models were detected (read count > 5) in at least one growth condition (Supplementary Table S2). The clustering analysis of individual replicates resulted in a tree with four major branches (Figure 1D). Mycelia grown on control MMN medium and medium reduced in PO_4_^−^ clustered together and close to the group of mycelia grown in a surplus of glucose or PO_4_^−^. Mycelium grown in the absence or surplus of NH_4_^+^ and in medium reduced in glucose clustered independently, indicating a dissimilar or unique transcriptomic response in these growth conditions. 

We identified gene groups showing unique responses to the different conditions by clustering similar transcriptomic patterns into 594 nodes using SHIN+GO (Figure S1; Supplementary Information). Supplementary Table S1A summarizes the mean expression pattern and other characteristics of condition-specific nodes. NH_4_^+^ availability induced specific and highly differential up-regulated expression of genes clustering in five different SOM nodes. Within the four nodes specific for an NH_4_^+^ surplus (i.e., nodes 325, 568, 576 and 579) genes encoding proteins with a predicted function in NH_4_^+^ transport, such as the AmT MeaA-like NH_4_^+^ transporter (prot ID 313221) in node 568 and an Rh-like protein NH_4_^+^ transporter in node 325, were found. Besides, genes encoding proteins with a function in growth and reproduction were specifically regulated by high NH_4_^+^ availability. Genes annotated with functions related to fungal mating-type pheromone (node 325), sugar transport and metabolism (nodes 568 and 576) and protein turnover (node 579) showed an expression specifically induced by profuse NH_4_^+^ next to many genes of unknown function. Reduced NH_4_^+^ concentration in the culture medium resulted in the specific up-regulation of 18 genes collected in node 352 and encoding a hydrophobin, two clitocypins (protease inhibitors), a fungal pheromone-related protein and several proteins of unknown function. Genes compiled in six SOM nodes (i.e., 487, 514, 525, 542, 571 and 572) showed an expression profile specific to reduced glucose availability. Limited glucose availability resulted in a negative differential transcription (>2 log2 fold difference) of these genes. Encoded functions included energy metabolism and conversion (aldehyde dehydrogenase in node 514), cellular signaling (protein kinases, G-protein subunit in nodes 487 and 571), transcription factors (bZIP and TEAD family transcription factor in nodes 525 and 572, respectively), detoxification (cytochrome P450 monooxygenase in node 525 and glutathione-S-transferase in node 572) and unknown. Two SOM nodes (569 and 570) clustered genes with a transcription pattern marked by a negative differential expression in response to both low NH_4_^+^ and glucose availability. In total, 15 out of the 19 genes that clustered in these two nodes and that showed this specific expression pattern encoded proteins of unknown function, two encoded a zinc finger protein, one a protein kinase and one a transaldolase.

SOM nodes clustering genes with a specific expression in response to an altered PO_4_^−^ concentration of the culture medium or excess glucose were not detected. In general, few genes (0–15) were significantly differentially expressed (>2 log2 fold change) under these conditions (Supplementary Table S2). When considering a lack of PO_4_^−^ in the culture medium, no genes were significantly differentially expressed compared to control MMN medium. Fifteen genes showed a significant differential gene expression in response to high PO_4_^−^-containing medium (Supplementary Table S1B). These genes were spread over nine different SOM nodes. Significantly down-regulated genes due to high PO_4_^−^ were also all regulated by the absence of NH_4_^+^ and reduced glucose availability. Genes significantly up-regulated by PO_4_^−^ were co-regulated by the presence of high glucose concentrations in the growth medium. Functions encoded by these genes did not include P_i_ (or polyP_i_) transport nor vacuolar polyphosphate (polyP) accumulation. Three polyP phosphatase-encoding genes predicted to be involved in polyP_i_–P_i_ conversions and vacuolar accumulation were regulated by high PO_4_^−^ availability to some extent (−0.61, 0.36 and 0.30 log2 fold change for protein id 317181, 589527 and 708028, respectively; Supplementary Table S2). Eleven genes not only were expressed at a higher level due to an increased glucose availability (+C) but also regulated by NH_4_^+^, PO_4_^−^ and/or reduced glucose availability. Supplementary Table S1B shows the fifteen most significantly regulated genes for each nutritional condition and includes 54 different genes, of which 65% (35/54) encode a protein of unknown function. A high proportion (22% or 12/54) of the most highly regulated genes under different nutritional conditions encoded for small secreted proteins (SSPs), all of which, except one hydrophobin (prot. ID 241509), were of unknown function and several of them (33% or 4/12) have been previously categorized as being mycorrhizal induced (MiSSP).

### 3.3. Time Course of Ectomycorrhizal Development 

**Figure 2:**
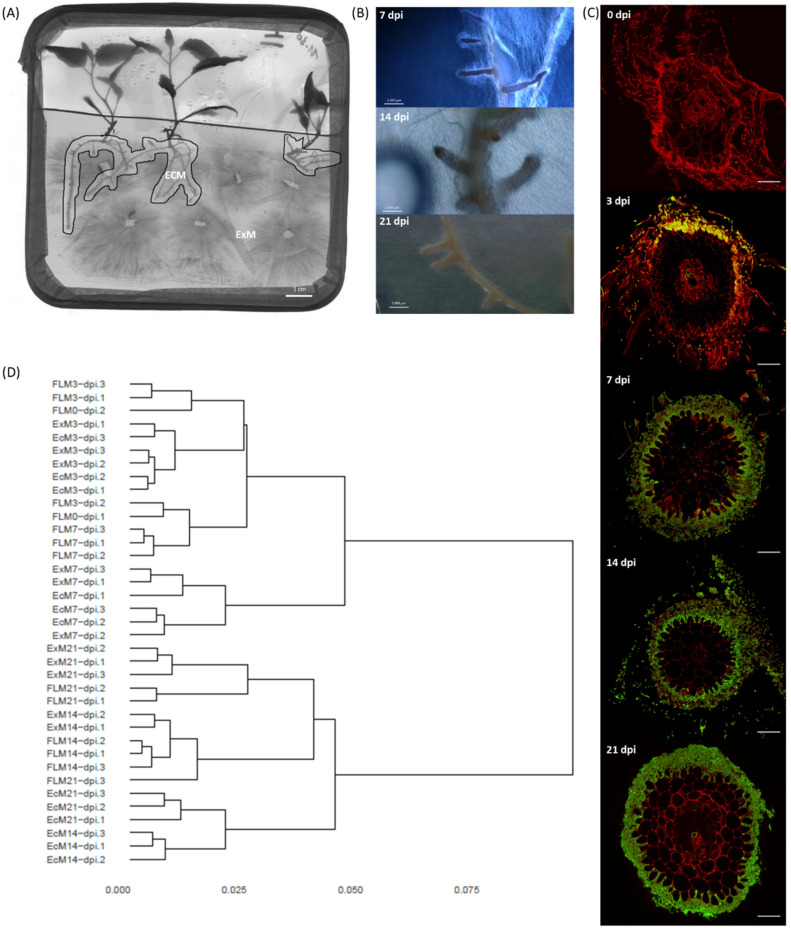
Responses of *L. bicolor* to *P. tremula x alba* at different time points post-inoculation. (**A**) In vitro co-culture of *Laccaria* and Poplar. Mycelium within a distance of 0.5 cm of a root was considered as ectomycorrhizal (EcM), mycelium at a longer distance as extramatrical (ExM). (**B**) External view of representative EcM root tips at different time points. (**C**) Transversal sections of lateral roots at 0, 3, 7, 14 and 21 dpi. Sections were stained with PI and WGA-oregon green to visualize plant (red) and fungal (green) cells, respectively. (**D**) Hierarchical clusters of overall transcriptomic responses of 37 biological replicates belonging to different stages of EcM development.

Three days post-inoculation, the first signs of *L. bicolor* mycelial attachment to *P. tremula x alba* roots became visible when using a petri dish in vitro system (Figure 2A–C). At that point, very few (0–5) short lateral roots were present and showed the start of fungal mantle development. Root hairs were still abundantly present as in non-inoculated lateral roots. One week post-inoculation, a fungal mantle surrounded all short lateral roots and *L. bicolor* had penetrated between the epidermal cells of *P. tremula x alba* roots. At two weeks post-inoculation, short roots were abundant and violet-colored due to the pigmentation of the fungal hyphae. *L. bicolor–P. tremula x alba* ectomycorrhiza were fully developed, with a thick, dense mantle and Hartig net. Three weeks post-inoculation, fungal hyphae formed a thinner but denser mantle. At this point, several individual epidermal root cells were fully surrounded by the fungal hyphae of the Hartig net. The roots were no longer violet-colored due to the disappearance of the pigment from the fungal hyphae.

### 3.4. Symbiosis-Regulated Transcriptome

The transcripts of 15,341 out of 23,130 different *L. bicolor* gene models were present in a read count higher than five in at least one developmental condition (i.e., free-living mycelium (FLM), extra-radicular mycelium (ExM) and ectomycorrhiza (EcM)). Gene models showing similar expression profiles were clustered into 621 SOM nodes (Supplementary Figure S2). The dendrogram based on the distances of gene expression similarity among all samples showed two major branches indicating a major difference in transcriptomic profiles between early (0, 3 and 7 dpi) and late (14 and 21 dpi) sample points (Figure 2D). At three and seven days post-inoculation, ExM and EcM samples had similar transcriptomes and clustered close to FLM transcriptomes. At seven days post-inoculation, ExM and EcM transcriptomes were slightly different from the other samples, as indicated by their separate clustering in the dendrogram. Two SOM nodes were identified as differentially expressed for ExM/EcM compared to FLM (>2 log2 fold change) at early time points (3 and 7 dpi). Putative functions associated with these genes are secondary metabolism (terpene synthase) and signal transduction (protein-tyrosine phosphatase, cyclin-like F-box) but also several oxidoreductases (dehydrogenases) and a nitropropane dioxygenase, reactive oxygen species detoxification (superoxide dismutase) and transcription (bZIP transcription factor). Most of these genes were also among the most regulated in *L. bicolor* in presence of the plant (ExM and EcM) at 3 and 7 dpi (Supplementary Table S1D). One SOM node (596) was identified as containing genes up-regulated uniquely in EcM at seven days post-inoculation. This node grouped seven gene models with two annotated as transporters of the Major Facilitator Superfamily, a protease, an esterase, a deoxy gluconate dehydrogenase a transcription factor and a peroxin (Supplementary Table S1C). However, none of these gene models were among the fifteen most regulated in EcM at this time point (Supplementary Table S1D).

At 14 and 21 days post-inoculation, EcM transcriptomes were similar to each other and clearly different from their ExM and FLM replicates (Figure 2D). Eight SOM nodes (9, 10, 11, 19, 26, 27, 53 and 385) grouped genes specifically regulated (>2 log2) in EcM samples 14 days post-inoculation (Supplementary Table S1C). The mean transcription level at 14 dpi (and 21 dpi for nodes 19, 26, 27 and 53) was above the 95th percentile of all genes included in the model except for one (node 385) of these SOM nodes. A large fraction (24% or 43/106) of the proteins encoded by the genes grouped in these nodes was of unknown function and not associated with any functional domain. Besides, a few genes previously annotated as MiSSPs (nodes 9, 11 and 385), a chitinase (node 9) and a glucanase (node 10), as well as many genes annotated as oxidoreductase, including several (flavo)cytochrome-related enzymes (cytochrome C oxidase, node 9; ferric reductase-like transmembrane component, node 10; cytochrome P450, nodes 11, 19, 26, 27 and 385), a DYP-type peroxidase and laccases (nodes 19 and 53), a proteinase inhibitor (clitocypin and proteinase inhibitor I9, node 19, highly enriched in node 27) and transport of nitrogen (H+/oligopeptide symporter, node 19; amino acid permease, nodes 27 and 53; ammonium transporter, node 385; major facilitator superfamily general substrate transporter, nodes 11, 19 and 26) or sugar (DUF6 nucleotide sugar transport related in node 19), were specifically regulated in EcM at 14 dpi. Genes with a putative function in signaling and protein–protein interactions (protein kinases, such as MEKK related protein kinase, NACHT nucleoside triphosphatase, WD40 repeat, Ankyrin and tetratricopeptide region) were identified in nodes 9, 10, 11 and 385. Some of the genes identified as having a unique expression pattern in EcM at late sample points (nodes 9, 10, 19, 27 and 385) were among the 15 most regulated for these conditions. However, most of the highest regulated genes in EcM at 14 and 21 dpi, such as MiSSP17 (prot ID 332226), gluconate transport inducing protein with gti1/pac2 domain (prot ID 603166), hydrophobin (prot ID 241509) and Rh-like ammonium transporter (prot ID 331747), were also highly regulated at early time points (3 and 7 days) in EcM only. MiSSP7 and 19 other previously identified MISSPs were mostly regulated to a lesser extent in EcM and were spread over different SOM nodes (Supplementary Table S3). However, for several of them, including the functionally characterized MiSSP7 and MiSSP8, an EcM- or EcM/ExM-specific expression profile was detected (Supplementary Figure S3). 

### 3.5. Transcriptomics of the Developing Basidiocarp

**Figure 3:**
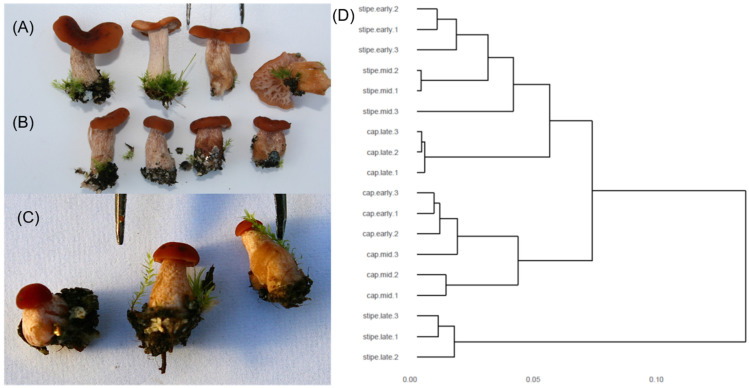
*L. bicolor* at different stages of basidiocarp development. (**A**–**C**) Images of basidiocarp specimen of early (**C**), medium (**B**) and late (**C**) stage development. (**D**) Hierarchical clusters of overall transcriptomic responses of 12 biological replicates belonging to different stages of basidiocarp development.

Three developmental stages of *L. bicolor* basidiocarp (Figure 3A–C), subsequently referred to as early (Figure 3C), medium (Figure 3B) and late (Figure 3A), were harvested under Douglas fir in a nursery bed and separated into cap and stipe before RNA extraction and sequencing. The transcripts of 16,609 out of 23,130 *L. bicolor* gene models were detected (read count > 5) in at least one basidiocarp developmental stage. The clustering analysis of individual replicates resulted in a tree with three major branches (Figure 3D). The transcriptomes of early- and middle-aged stipes clustered together as well as early- and middle-aged cap transcriptomes. Late-stipe transcriptomes formed a third branch, while late-cap transcriptomes were located between early–medium stipes and caps. ShinGO was run and the clustered genes showed similar transcriptomic patterns into 644 SOM (Self Organizing Map) nodes (Figure S4). For 22 nodes, genes were up-regulated in late caps compared to late stipes (Supplementary Table S4), while only two nodes (476 and 504) with genes up-regulated in the middle stipe compared to cap were identified. Some of the nodes with late-cap versus -stipe up-regulated genes also contained genes with differential expression between cap (late versus middle or early cap) or stipe stages (early or middle versus late), indicating an overall higher expression in the cap, but also a regulation during development in both cap and stipe. We further hierarchically clustered the 6070 genes found to be differentially expressed between at least two basidiocarp developmental stages (Figure S5A, Supplementary Table S6) and identified eight gene clusters with distinct expression patterns in the developmental stages. Overall, early- and middle-cap stages clustered together as well as early- and middle-stipe stages, while late-cap and -stipe stages showed very distinct expression patterns (Figure S5A). Within the blue cluster with a gene expression peak in early and medium cap, we found the highest number of genes with functions related to cell cycle (for example, cyclins, septin, M-phase_inducer_phosphatase), as well as many SSPs (hydrophobin, cupredoxin and cysteine-rich proteins, but most of them were of unknown function). The highest percentage of SSPs, but also of CAZymes (for example GH18 chitinases, expansins, GH16 lectins), proteases and lipases, are found in the magenta cluster with gene expression peaking in late cap (Figure S5B), indicating an overall high secretome activity, probably linked to the mature, fully developed mushroom. A high number of CAZymes (3%) were also found in the turquoise cluster with expression peaks in early stipe and cap (GH16, CE4, AA1, -3, -5 and -6) and in the red cluster with expression peaks in late stipes (GH18, GH5 and AA5) (Figure S5A,B). In the yellow cluster with gene expression peaks in both early and medium cap and stipes, genes coding for metabolic processes (lipid, nitrogen, malate and polysaccharide) were enriched (Figure S5C). In another cluster linked to early stipe and cap (turquoise), we found further enrichment of genes coding for metabolism (glutamate, alcohol, aromatic compounds) but also transport, in particular, carbohydrate transport. In early and medium cap (blue cluster), genes with functions in protein biosynthesis (translation, ribosome biogenesis, protein folding and elongation) but also transcription (nucleosome and mRNA metabolism) were further enriched, indicating a high cellular activity, probably due to reproduction processes, including meiosis and mitosis. Clusters containing genes with expression peaks in late cap (magenta), late stipe (red) or both late cap and stipe (green, orange) were enriched for defense-related GO categories, but also chitin catabolism, cell wall macromolecule catabolism, glycolytic processes, spermine/spermidine biosynthesis and response to pH and are probably linked to the final maturation of the basidiocarp but also defense against insects and other small animals feeding on the mushroom (Figure S5C).

### 3.6. Similarities and Dissimilarities between Transcriptomes of Different Morphological and Functional L. bicolor Structures 

**Figure 4:**
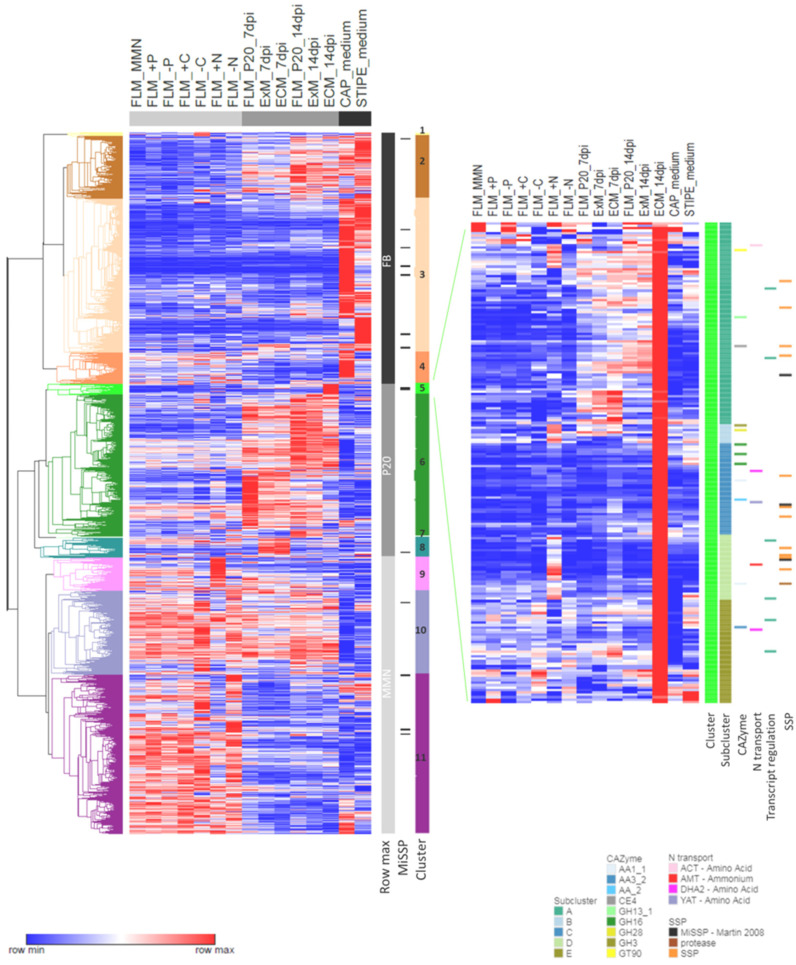
Hierarchical clustering of 23,293 genes expressed in different functional and morphological structures of *L. bicolor*. Each row represents a gene model. The heatmap shows the gene expression level normalized to the row maximum. In total, 3 identified gene groups were differentially expressed throughout the different experimental conditions and 11 clusters were identified based on specific expression profiles. Cluster 5 groups genes with a maximum expression level in mature mycorrhiza and can be divided into subclusters A–E based on their co-regulation under different conditions. Genes with functions in N transport, transcript regulation, CAZymes and SSPs are highlighted.

The transcription profiles of 23,293 gene models expressed with a minimum expression level of 5 RPKM in one of the included experimental conditions were visualized and compared for FLM grown in different nutritional conditions, ExM and EcM at 7 and 14 dpi, and basidiocarp (or fruiting body, FB) caps and stipes of medium age. Overall, hierarchical clustering revealed that the gene models were divided into three major groups based on the condition of maximal expression (Figure 4). All genes collected in the first group showed a maximum expression level in FB (cap or stipe) and were subdivided into three clusters based on the expression level in other conditions. In particular, gene models of cluster 3 were highly regulated in FB compared to all other conditions. Nevertheless, some of the gene models included in this cluster were regulated >2-fold by other conditions. For instance, ten SSPs, previously annotated as MiSSP [10], were found in cluster 3. In addition, in this study, seven out of these SSPs were regulated to some extent in EcM and/or ExM at least one time point of EcM development. Two (prot ID 333197 and 395272) were expressed below the limit of 5 RPKM in all mycelia grown on P20 (Figure S3).

The gene models compiled in the second group have their expression maximum in one of the mycelia grown on P20 medium and were divided into four clusters (5–8) based on the particular condition showing the highest expression level (Figure 4, Supplementary Table S5). Gene models grouped in cluster 5 showed their expression maximum in EcM at 14 dpi and can be divided into six subclusters (5.1–5.6). In general, gene models included in cluster 5 were relatively lowly expressed in most other conditions; therefore, they were of particular interest when studying EcM morphology and functioning. Cluster 5 contained 15 SSPs; among them, there were MiSSP7 and two other MiSSPs previously identified by [10]. Only four of the SSPs in cluster 5 were not regulated by any other condition. Most of the others, including MiSSP7 (subcluster A), were regulated to some extent by NH_4_^+^ (+N). Five gene models encoding CAZymes (three GH16, one AA1_1 and one AA2) were part of the EcM 14 dpi specific subcluster 5.3, but several others were co-regulated by the presence of NH_4_^+^ (GT90, GH13.1, GH3, GH28 and AA1.1). Two amino acid transporters were found in subcluster 5.3. Other transporters involved in N transport (e.g., AMT ammonium permease) included in cluster 5 were also regulated by NH_4_^+^ availability (presence or absence). Furthermore, gene models co-regulated by NH_4_^+^ availability or other nutritional conditions (e.g., –C in subcluster 5.5) included several proteins involved in transcriptional regulation. EcM 14 dpi specific subcluster 5.3 contained 73 gene models encoding (additionally to SSPs, CAZymes and N transport) a sugar and Cu transporter, a Rab GTPase activator, several oxidoreductases (FAD or heme binding) and genes of unknown function (Supplementary Table S4). Gene models in cluster 6 and 7 shared a high relative expression in multiple of the P20-grown mycelia (Figure 4). Cluster 8 grouped gene models with the highest expression level at early time points of EcM development (EcM and ExM 7 dpi). Genes in this cluster were of particular interest for the understanding of structural and metabolic changes in mycelial colonies and are required for EcM development. Many of the genes in this cluster responded similarly in FLM upon changes in nutrient availability and a relatively small group of 67 gene models showed a contrasting expression pattern when compared to all other conditions assessed. Within this group, several genes encoding proteins with a predicted redox activity and functions in reactive oxygen metabolism or signaling (e.g., protein phosphatase and kinase) were found. However, interestingly, no CAZymes and few SSPs were found in this group of 67 genes; therefore, this highly contrasted with the group of genes identified to be specifically up-regulated in mature EcM at 14 dpi.

Cluster 3 grouped gene models with maximum expression level when grown on MMN medium (control or nutritionally modified) and was more similar to the P20 group than to the FB group (Figure 4). Within the MMN group, three clusters (9–10–11) were defined. The transcription profile of the gene models included in cluster 9 was characterized by a high expression due to elevated levels of NH_4_^+^ in the medium (+N). Cluster 10 and 11 grouped gene models showing a high relative expression in many of the MMN-grown mycelia, which could be almost equally highly expressed in many (cluster 10) or few (cluster 11) P20-related conditions. These clusters contained six SSPs previously annotated as MiSSP (Martin et al., 2008), four of which were induced by mycorrhization (EcM 7 and/or 14 dpi) in this study (Figure S3).

An overall comparison of the genes up-regulated in EcM at 14dpi and all basidiocarp stages together (FB) with FLM at 14 dpi (fold change >log2 1, FDR *p*-value < 0.05) identified 308 transcripts that were only up-regulated in EcM, 5073 only up-regulated in FB and 668 transcripts up-regulated in both EcM and FB (Figure S6A, Supplementary Table S7). Overall, we found, among the FB-only up-regulated transcripts, more genes of unknown function and KOG classified as Information/Storage/Processing (Figure S6B). Among the EcM-only up-regulated transcripts, genes coding for transporters as well as for transcription factors were more frequent than in FB-only and among the EcM and FB up-regulated transcripts. The comparison confirmed that many MiSSPs likely play a role in both EcM formation but also in basidiocarp development (Figure S6B). The EcM-only induced transcripts were enriched for the GO term transport and, in particular, carbohydrate transport, but also for the GO term defense response (Figure S6C). The EcM and FB up-regulated transcripts were also enriched for transport, including amino acid, potassium ion and sulfate transport, but also for cell wall macromolecule catabolic processes, probably reflecting the cell wall remodeling necessary, in both cases, to form new tissues. Among these transcripts, we found transcripts coding for clitocypin cysteine proteinase inhibitor (prot ID 293826, 318727) or MiSSP8, both previously identified as highly EcM- and FB-up-regulated [10]. As already shown for the different basidiocarp stages and tissues, FB-only up-regulated transcripts showed an enrichment for the GO term metabolic process, in particular, carbohydrate, one-carbon and nucleoside metabolism. A role of aquaporins has previously been suggested for both *L.bicolor* EcM [31] and FB [32] development. Interestingly, we found that two aquaporins (456764 and 443240) were FB-only up-regulated, while another aquaporin (671860) was EcM-only induced compared to the expression in FLM.

## 4. Discussion

### 4.1. L. bicolor PO_4_^−^ Homeostatic Range Is Larger Than NH_4_^+^ Homeostatic Range

Mycorrhizal fungi are known for their ability to efficiently forage soils for any available Pi, to incorporate it into NTPs (nucleoside triphosphates), subsequently polymerize it into polyP and accumulate massive amounts of this polyP into tubular vacuoles for translocation towards the host [33,34,35]. Uptake, storage and remobilization of Pi in ectomycorrhizal fungi is well studied at the physiological level and likely requires the coordinated functioning of several transporters and enzymes to fine-tune Pi metabolism in response to external Pi concentrations [36,37,38,39,40,41]. In the EcM fungi *Hebeloma cylindrosporum*, *Boletus edulis* and *Tricholoma* sp., Pi deficiency results in an up-regulation of the gene expression of plasma membrane-localized H^+^: Pi symporters of the Pht1 gene family, which is involved in Pi uptake. The *L. bicolor* genome encodes five transporters of the Pht1 family with a putative function in Pi transport [10]. None of these transporters were transcriptionally regulated in response to growth for one week in a wide range (0–25 mM) of external Pi concentrations, neither were Vacuolar Transporter Complex (Vtc) subunits, suspected to be involved in polyP synthesis, nor were putative polyphosphatases and tonoplast localized efflux proteins, which are assumed to be necessary for Pi remobilization. Nevertheless, mycelial P concentrations reflected well external Pi concentrations in *L. bicolor* without any visible effect on biomass production and only a subtle effect on the expression of 15 (out of 23130) gene models mainly encoding proteins of unknown function in case of Pi excess. This suggests an efficient homeostatic system, eventually including shuttling of Pi towards the vacuole to overcome unspecific binding in case of excess and a remobilization in case of deficiency to allow ATP metabolism and synthesis of biomolecules (e.g., DNA synthesis) to be performed in order to support growth. Pi homeostasis in *L. bicolor* and the different transporters and enzymes involved, might be regulated mostly at the post-transcriptional or post-translational level, as is suggested for the Vacuolar Transporter Complex subunit 2 (Vtc2) of *Saccharomyces cerevisiae* which localizes differentially (tonoplast–plasma membrane/nucleus), depending on Pi availability [42]. Differences in the regulation level (transcriptional vs. post-translational) might result in differences in response time to external conditions and contribute to interspecific differences in Pi uptake kinetics, reported previously for EcM species [43]. Alternatively, though less likely, transcriptional response of Pi homeostatic genes might be induced transiently immediately upon changing external concentrations, or a critical low level of internal Pi, not yet reached in this study, might be needed. *L. bicolor* cultures were maintained on P5 medium and pre-cultured on MMN, both relatively rich in Pi and consenting mass storage, whereas Ref. [44] used a culture medium with a factor 2.5 lower Pi content to culture *H. cylindrosporum* awaiting experimentation. A thorough functional characterization including protein modifications and kinetics of transporters and enzymes putatively involved in Pi homeostasis is required to shed light on the functioning of this efficient homeostatic system in *L. bicolor*, its role in adaptation towards forest soils and its contribution to the ecology of *L. bicolor*.

The NH_4_^+^ homeostatic range of *L. bicolor* is smaller than the one observed for PO_4_^−^ and reflects its adaptation to forest soils. Both growth in absence and surplus of NH_4_^+^ induced major differences in gene expression and tended to affect mycelial growth (Figure 1). In forest soils, nitrogen stored in proteins and complex sugar derivatives accumulate to a high concentration, but water soluble NH_4_^+^ is scarce. Yet, NH_4_^+^ is the most energetically favorable and the preferred nitrogen source of ectomycorrhizal fungi, compared to more abundantly present organic nitrogen [34]. The assimilation route of NH_4_^+^ into glutamate and/or glutamine used by *Laccaria* requires the presence of sufficient carbon to support amino acid synthesis [45]. Therefore, NH_4_^+^ assimilation and carbon metabolism are expected to be interwoven and partially co-regulated. We could not identify marker genes specific for an NH_4_^+^ deprivation response (Supplementary Table S1A). Though two clusters of genes were specifically down-regulated upon NH_4_^+^ deprivation and growth in sugar-reduced medium. These clusters, including two transcription factors, a protein kinase, a transaldolase and many genes of unknown function, might group genes with a function in the regulation of growth and primary metabolism as an adaptive response to NH_4_^+^ and carbon availability. Shared transcriptional responses for NH_4_^+^ and glucose deprivation were found previously for the mycorrhizal ascomycete *Tuber borchii* [46]. Besides, reduced glucose availability resulted in a down-regulation of several other genes, including many with a predicted function in signaling, gene regulation or the release of nitrogen from organic sources (e.g., chitinase, quinoprotein amine dehydrogenase, adenosine deaminase and aspartic type endopeptidase) in *L. bicolor* (Supplementary Table S1A). The response of these genes was not shared with any of the other tested growth conditions. Together, this indicates the importance of glucose as the primary carbon source for ectomycorrhizal metabolism and strengthens the hypothesis of ectomycorrhizal fungi degrading complex organic material in an attempt to scavenge nitrogen rather than carbon [47].

**Figure 5:**
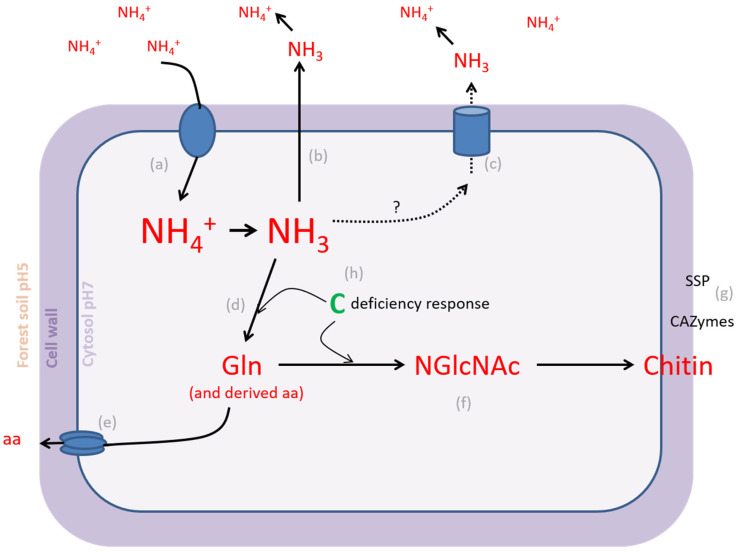
Schematic overview of transcriptional changes and potentially associated cellular responses to excess external NH_4_^+^ in *L.bicolor*. (**a**) NH_4_^+^ likely enters cells using unspecific transporters. In the cytoplasm, a fraction of the NH_4_^+^ turns into NH_3_ due to a pH shift. (**b**) Gaseous NH_3_ partially diffuses out of the cell and (**c**) active transport may contribute to NH_3_ efflux. In the soil, most NH_3_ is protonated again. (**d**) Another part of cellular NH_3_ is assimilated in gln and derived aa (**e**), which may be transported out of the cell. (**f**) Starting from gln, NGlcNAc and subsequently chitin are synthesized. (**g**) CAZymes assist in cell wall remodeling. SSPs may also contribute to cell wall remodeling or cell–cell communication. (**h**) Excessive assimilation of NH_3_ requires carbon and results in a carbon deficiency response. Part of these responses (**b**,**c**,**e**,**g**) are shared with mycelium in mature mycorrhiza at 14 dpi. gln = glutamine; aa = amino acids; NGlcNAc = N-acetylglucosamine; CAZyme = Carbohydrate Active Enzyme; SSP = small secreted protein.

Excess extracellular NH_4_^+^ is toxic through the high energetic cost of futile transmembrane cycling and disruption of the transmembrane pH gradient, which leads to apoptosis in many organisms [48]. However, the fungal model species *Saccharomyces cerevisiae* can withstand temporal high external NH_4_^+^ concentrations through the effective regulation of NH_4_^+^ importers and adaptive growth response. The NH_4_^+^ transceptor Mep2, an NH_4_^+^ permease from the Amt/Mep/Rh family with sensing function, is required for the transition to filamentous growth in response to low NH_4_^+^ conditions. Mep2 activity is regulated by phosphorylation. Under nitrogen-sufficient conditions, this transporter is not phosphorylated and present in an inactive conformation. Under high external NH_4_^+^ concentrations, it is degraded and transcription is repressed [49]. The constitutive overexpression of yeast ammonium permeases Mep1–3 results in an NH_4_^+^ toxicity response. Even so, high external NH_4_^+^ induces a toxic response by passive leakage trough K^+^ channels at low K^+^ availability. In both situations yeast cells excrete amino acids [50]. A release of amino acids in the medium was detected previously for *L. bicolor* after a period of exponential growth in the presence of inorganic nitrogen [51]. Surprisingly, the gene expression of two Rh-like ammonium transporters was increased in the presence of high external NH_4_^+^ concentrations in *L. bicolor*. One of these transporters was the homologue of the *H. cylindrosporum* low-affinity NH_4_^+^ transporter HcAMT3, which was shown to be down-regulated by NH_4_^+^ [52]. The ammonium transporter (Amt) gene family is expanded in *L. bicolor* when compared to *H. cylindrosporum* and fungi with saprobic or parasitic lifestyles [53]. Gene duplication, followed by neofunctionalization, might have resulted in an altered function for the identified highly expressed Rh-like ammonium transporter genes in *L. bicolor*. Rh-like ammonium transporters facilitating the diffusion of gaseous NH_3_ were previously identified in prokaryotes and invertebrates and are involved in the excretion of NH_3_ when the supply of exogenous nitrogen is high [54,55]. The presence of a GPR1/FUN34/yaah family-encoding gene, with typical signature of an NH_3_ transporter such as the yeast Ato3 (ammonia transporter outward) member, characterized by Ref. [56] in a gene cluster specifically up-regulated by high external NH_4_^+^ concentrations, further supports the hypothesis of NH_3_ excretion by *L. bicolor*. Alternatively, excess nitrogen could be stored in complex sugar derivatives such as the cell wall polymer chitin and recuperated when external nitrogen availability is low (Figure 5). Many ectomycorrhizal fungi show N-acetylglucasaminidase activity, a functional trait of chitin degradation in response to nitrogen deprivation [57]. In *L. bicolor,* several acetylglucosaminyltransferase (N-GlcNAc transferase)-encoding genes were specifically up-regulated as a response to excess NH_4_^+^. N-GlcNAc transferases are involved in protein glycosylation and chitin biosynthesis pathways [58]. Besides several sugar transporters, CAZymes, including cell wall remodeling enzymes, were specifically up-regulated in *L. bicolor* mycelium exposed to high external NH_4_^+^ levels. A model for cellular nitrogen homeostasis and assimilation in *L. bicolor* is represented in Figure 5.

As in pathogenic fungi, ammonium homeostasis and regulation of mating-type loci seems to be linked in *L. bicolor*. Mating-type pheromone and pheromone receptor genes showed an increased expression when mycelium was grown in the presence of excess NH_4_^+^. In *Ustilago maydis* and *Cryptococcus neoformans,* mating pathway and pathogenesis are regulated in response to external ammonium supply through the action of Amt family transporters with an ammonium-sensing function (transceptors—[49,59]). The functional characterization of *L. bicolor* putative Amt family transporters is needed to confirm the role of sensor and explore their role in nutrient signaling.

### 4.2. EcM Development Is a Two-Step Process

**Figure 6:**
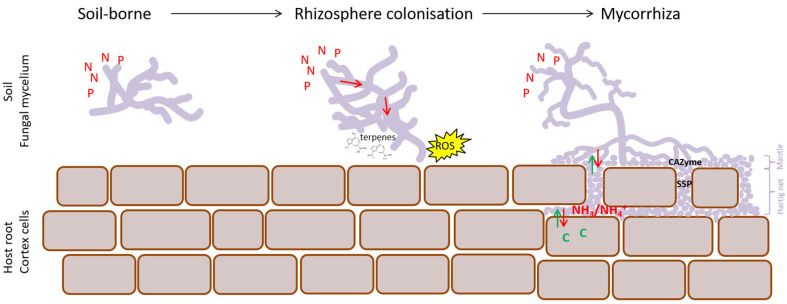
Dynamics of EcM development. Transition from soil-borne mycelium towards functional symbiosis requires a two-step transcriptional reprogramming. Initial contact with a plant host, when entering the rhizosphere, marks the first step and results in priming of the mycelium with roles suggested for ROS signaling and terpenes. The second step corresponds to functional differentiation between ExM and EcM with differential expression of CAZymes, SSPs, nitrogen (amino acid and ammonium) and sugar transporters. At this point, both fungal mantle and Hartig net are well-developed and visible. ROS = reactive oxygen species; SSP = small secreted protein; aa = amino acid.

Morphogenesis and maintenance of pine ectomycorrhiza is a dynamic process marked by waves of transcriptional reprogramming in *H. cylindrosporum* [60] and *Lactarius deliciosus* [61]. This seems to be the same for *L. bicolor* when colonizing poplar roots, with particular gene clusters (Supplementary Table S1C) being induced upon early contact with the host tree and normalizing again when morphogenesis progresses. Other clusters were popping up at the start of mantle formation (3 dpi); they showed an expression peak in well-functioning mature mycorrhiza (14 dpi) and eventually showed a relative decline in expression with aging (21 dpi). EcM development entailed a two-step process, with a clear separation of early and late events (Figure 6). However, surprisingly, gene clusters specifically induced during early morphogenesis events, i.e., initial contact and mantle formation (3–7 dpi), were also all induced in extramatrical mycelium. This might be attributed to a systemic response of the fungus to the presence of the plant. It is likely that mycelium in the vicinity of the plant detects some plant signal and transduces it to the rest of the colony to prime it for symbiosis. Alternatively, plant volatile signals might concentrate in the closed petri dish system and directly influence extramatrical mycelium at a relatively large distance from the roots. Similarly, CO_2_ produced by fungi, which accumulates in such closed petri dishes, leads to increased plant biomass production [62]. However, this is less likely, since transcriptomes of extramatrical mycelium cannot be distinguished from those of free-living mycelium at other time points. Genes with a putative function in signaling (e.g., cyclin-like F box), gene regulation (bZIP transcription factor) and reactive oxygen metabolism (e.g., superoxide dismutase, peroxisomal biogenesis protein and several dehydrogenases) were ubiquitous in early induced clusters, as well as genes encoding unknown functions (Supplementary Table S1C). An induced stress response, including anti-oxidative enzymes (e.g., superoxide dismutases), was previously observed in developing ectomycorrhiza for several fungal species and hypothesized to act as defense mechanisms against the root oxidative burst [60,63,64]. However, current data suggest an additional role for reactive oxygen species as long-distance signaling molecules and they might be needed to induce structural changes in the fungal colony to prepare for effective nutrient translocation and compatible interaction with the plant host. It has been previously suggested that the activation of oxidative stress pathways is a possible mechanism used by EcM fungi to mediate host specificity [65]. The presence of a peroxisomal biogenesis protein, short-chain dehydrogenase, esterase and taurine catabolism dioxygenase could be an indication of increased fatty-acid metabolism and beta oxidation to meet energy requirements or reactive oxygen species production. An increase in beta oxidation, compared to mature mycorrhiza, characterizes *P. involutus* rhizomorphs when associated with birch [66]. Besides, two genes predicted to encode terpene synthase were specifically and transiently induced upon early contact with poplar (3–7 dpi) in *L. bicolor* emerging mycorrhiza and extramatrical mycelium. This could illustrate an effective chemical communication between both mutualistic partners. Sesquiterpenes produced by ectomycorrhizal fungi were identified previously as volatiles triggering lateral root production in host and non-host plants [67].

Five gene clusters were identified as specific for mature *L. bicolor–P. tremula x alba* mycorrhiza and contained genes with an expression peak at 14 dpi, though most of them were regulated to a certain extent throughout the whole process of morphogenesis (3–21 dpi; Supplementary Table S1C). These clusters likely contain genes encoding key functions in development and maintenance of mycorrhiza, including CAZymes potentially involved in fungal and plant cell wall remodeling (e.g., multicopper oxidase, chitinase) and many redox-related enzymes potentially involved in the detoxification (e.g., CYP450, Glutathione-S-transferase) of organic radicals. A reduced CAZyme gene content, but expressed during mutualistic interaction, is a general trait of ectomycorrhizal fungal genomes [6]. It has been hypothesized that the remaining CAZyme-encoding genes are deployed in cell wall remodeling during Hartig net formation rather than decomposition as an adaptation to the mutualistic lifestyle [18,60,68]. In these ectomycorrhizal-specific gene clusters, the presence of nutrient transporters seems obvious, taking into account the function in nutrient exchange between both mutualistic partners. We identified a member of the Major Facilitator Superfamily transporters, an amino acid permease and a Rh-like ammonium transporter, as highly and specifically regulated in mature mycorrhiza and confirmed the results of previous studies reporting an increased expression of nutrient transport proteins in ectomycorrhizal root tips [60,61,63,64,69,70]. Genes encoding proteins with a predicted function in protein–protein interaction (e.g., WD40 repeat, NACHT domain), posttranslational modification (e.g., protein kinase) and SSPs with a potential function in fungal–plant signaling are ubiquitous in mature ectomycorrhizal specific gene clusters. However, several MiSSPs, identified previously by [10] and including MiSSP7, which was characterized as a fungal effector acting on host plant jasmonic acid-regulated pathways, [13] were not detected as mycorrhiza-specific in the current analysis. This might be due to our sampling strategy, considering all mycelium within a 0.5 cm distance from the root as mycorrhizal and strongly diluting the signal of genes expressed at a very specific location, such as the Hartig net. Indeed, though to a lesser extent and not passing the threshold for specificity, previously identified MiSSPs [10] in greenhouse experiments were induced in mycorrhiza in this study (Figure S3). The use of a controlled in vitro system to study mycorrhizal development reduces the false positive detection rate. However, an in situ analysis of gene expression (or laser microdissection) in in vitro mycorrhiza is needed to distinguish between previously identified genes showing a high expression in greenhouse mycorrhiza due to local expression or due to co-founding environmental factors and to reduce the false negative detection rate in the in vitro system.

Three clusters of genes were identified as specific for mature mycorrhiza. They showed an expression peak at 14 dpi and a relative decrease afterwards (21 dpi) due to an increasing expression in free-living mycelium and extramatrical mycelium with age. One of these clusters mainly contained genes encoding protease inhibitors (e.g., clitocypin), the other two contain transporter proteins and redox-related enzymes (e.g., CYP450, dyp-type peroxidase) among other functions (Supplementary Table S1C). All included functions were detected previously as induced in ectomycorrhizal root tips of several fungal species [6,60,63,66,71]. However, specific genes might be related, rather, to senescence or colonial aging and function in nutrient and resource recycling rather than in mutualism. However, a detailed functional analysis of individual genes is needed to confirm this hypothesis. 

In conclusion, we present a model for EcM development (Figure 6). This is clearly a dynamic process involving a first step of systemic transcriptional re-programming in the fungal mycelium when entering the rhizosphere of a plant host. At this point, transcriptional responses of extramatrical mycelium and mycelium surrounding root tips are not differentiated and indicate priming of the mycelium for symbiosis with roles in signaling suggested for ROS and terpenes. A second step of transcriptional reprogramming marks the differentiation between extramatricular mycelium and functional EcM. At this point, transporters (mainly N and C) are highly differentially regulated as are MiSSPs and CAZymes acting on plant and fungal cell walls.

### 4.3. L. bicolor Mushroom Development Shares Transcriptomic Signatures with Other Agaricomyctes

The development of Agaricomycetes mushrooms represents a transition from simple to complex multicellularity and has been studied on the molecular level mainly in a few systems where fruiting is inducible under laboratory conditions (e.g., *Coprinopsis cinerea* [72], *Schizophyllum commune* [73] and, more recently, in *Agaricus bisporus* [74], *Armillaria* [75], *Agrocybe aegerita* [76,77], *Lentinus tigrinus* [78] and *Flammulina filiformis* [79]). These advances allowed researchers to adopt a comparative transcriptomic approach using six species [80] and to identify 300 conserved gene families involved in basidiocarp development. Genes related to fungal cell wall remodeling, targeted protein degradation, signal transduction, adhesion and SSPs were also identified for *L. bicolor* mushroom formation. Even though the earliest *L. bicolor* basidiocarp stage we sampled already had cap and stipe clearly developed (Figure 3C), we found known primordia-related transcription factors (TFs), such as bwc2 or c2h2, up-regulated in the early stage (cap and stipe). In addition, Hom1, which is involved in mushroom-tissue formation, was up-regulated in all basidiocarp stages compared to FLM. Further, transcripts known to be involved in *C. cinerea* mushroom signaling, maturation and elongation, such as dst2, eln3, or eln3-like, were conserved in *L. bicolor* basidiocarp development. If we take into account that our *L. bicolor* mushrooms were harvested from non-sterile environments, then the 1097 fruiting body development-regulated transcripts shared with *C. cinerea* (from, in total, 7475 developmentally regulated genes from [80]; see Supplementary Table S8) confirmed a highly conserved fruiting-related gene set.

### 4.4. Nutrients Are Key in Functional Differentiation of EcM Fungi

The main aim of this study is to understand how EcM fungi use their gene set and to decipher the principles and mechanisms by which gene activity orchestrates the development of symbiotic and reproductive structures. Gene clustering allowed us to identify co-expressed gene modules corresponding to a specific morphological structure or state. As for other multicellular organisms, few genes have a function restricted to one particular condition and are silenced in any other condition [81,82]. Transcripts for most of the genes in *L. bicolor* could be detected in multiple conditions and morphological and functional differentiation seemed to be the result of coordinated regulation of gene modules (Figure 4).

Genes with a function within the GO category cellular transport were highly regulated in all conditions. Perhaps, this is not surprising, since different morphological structures within the fungal colony have different functions in nutrient transport and homeostasis [83]. A role for nutrients as signals in developmental processes and, e.g., establishment and maintenance of symbiosis was expected. the co-regulation of a gene module, containing genes with a function beyond nutrient transport and metabolism, in both mature mycorrhiza and mycelium exposed to excess NH_4_^+^ (+N) identified nitrogen as a candidate for nutrient signaling in maintenance of the symbiosis. The induction of MiSSPs, including MiSSP7, with a demonstrated function in fungal–plant host interaction [13] by NH_4_^+^, suggested nitrogen availability to prime the mycelium for effective interaction with the plant host. Phosphate availability, on the other hand, induced only minor transcriptional changes in *L. bicolor* and co-regulation with symbiotic or reproductive structures was not detected. This is in line with previous findings for *P. involutus*, where Pi starvation and EcM development had independent effects on the transcriptome [84].

Transcriptomic data, as presented here, are aimed to put forward working models for further functional validation. It is clear, from this study, that processes underlying functional and morphological differentiation within the EcM fungal colony involve both conservative and adaptive processes. Basidiocarp development in *L. bicolor* shares transcriptomic signatures with other basidiomycetes and suggests mushroom development to be a conservative process. Some of the genes highly induced in basidiocarps are part of a module of genes up-regulated to a minor extent in mature mycorrhiza too and might function in pseudoparenchym formation or maintenance. In this way, genes involved in EcM development might be partly conserved among different species. However, other transcriptomic signatures underlying the establishment and maintenance of symbiotic structures and interactions with a plant host, such as regulation of GTP and ROS signaling, might also be conserved. We detected similarities and differences in responses to symbiosis development with previously published analyses focusing on *Suillus* sp. [71], *Pisolithus* sp. [85], *Lactarius* sp. [61], *H. cylindrosporum* [60] and *P. involutus* [84]. A higher spatio-temporal resolution of transcriptomic data within these species is required to decipher to which extent evolution of ectomycorrhizal symbiosis is convergent at the molecular level. However, responses to nutrient availability seem to be different among EcM fungal species with different species having different homeostatic ranges, as illustrated by the fact that *L. bicolor* does not transcriptionally respond to changes in Pi availability, whereas *P. involutus* clearly does [84]. Such adaptive responses in nutrient homeostasis might underly the resilience of the symbiosis and determine the success of a particular species in a particular environment. In conclusion, we used the current transcriptome atlas to generate hypotheses regarding the differentiation within a fungal colony and development of symbiotic and reproductive structures that should be tested experimentally. This transcriptome atlas for the ectomycorrhizal fungus *L. bicolor* will be a valuable resource to assess the resilience of the symbiosis and adaptive potential of EcM fungi upon environmental or genetic perturbations.

## Data Availability

The GEO accession numbers of RNA-seq reads at NCBI GEO database (https://www.ncbi.nlm.nih.gov/geo/, accessed on 10 December 2021) are as follows: GSE190443–GSE190445.

## Supplementary Materials

The following are available online at https://www.mdpi.com/article/10.3390/microorganisms9122612/s1, Supplementary Information, Figure S1: Transcriptomic response of *L. bicolor* on different nutritional conditions, Figure S2: Transcriptomic response of *L. bicolor* at different time points of single culture (FLM) and co-culture with poplar in ectomycorrhizal (EcM) and extrametrical mycelium (ExM), Figure S3: Expression profiles of previously identified MiSSPs, Figure S4: Transcriptomic response of *L. bicolor* at different stages of sporocarp development, Figure S5: Differentially expressed genes in early, middle and late stage caps and stipes of *L. bicolor* basidiocarps, Figure S6: Comparison of ectomycorrhiza (EcM) and fruiting body (FB) up-regulated transcripts compared to free-living mycelium (FLM), Table S1: Condition specific SOM nodes and top regulated genes, Table S2: SOM nodes of nutrition regulated transcriptome, Table S3: SOM nodes of symbiosis regulated transcriptome, Table S4: SOM nodes of basidiocarp development, Table S5: Hierarchical clusters of differential expressed genes from Figure 4, Table S6: List of fruiting regulated transcripts from Figure S5, Table S7: List of regulated genes from Figure S6, Table S8: Basidiocarp developmental regulated genes from Kriznan et al. 2019.
