# Supplementary material for: A Transcriptomic Atlas of the Ectomycorrhizal Fungus Laccaria bicolor
Source: Microorganisms. 2021 Dec 17;9(12):2612. doi: 10.3390/microorganisms9122612 (PMC8708209; doi:10.3390/microorganisms9122612)
Supplement: Supplementary file 1 [file microorganisms-09-02612-s001.zip › microorganisms-1464646-supplementary/Captions_Figures and tables.pdf]

Figure captions

Figure S1. Transcriptomic response of *L. bicolor* on different nutritional conditions. (A) Averaged transcriptomic profiles per condition. Vertical bars indicate transcription level. (B) Condition specific nodes.

Figure S2. Transcriptomic response of *L. bicolor* at different time points of single culture (FLM) and co-culture with poplar in ectomycorrhizal (EcM) and extrametrical mycelium (ExM). (A) Averaged transcriptomic profiles per condition. Vertical bars indicate transcription level. (B) Condition specific nodes.

Figure S3. Expression profiles of previously identified MiSSPs. (A) Transcription levels at different time points (0,3,7,14,21 days) post-inoculation in extramatricular mycelium (EXT), mycelium surrounding poplar roots (ECM) or mycelium grown in absence of a poplar host (FLM). (B) Normalized gene expression level upon growth in different nutritional conditions and in different functional and morphological structures.

Figure S4 Transcriptomic response of *L. bicolor* at different stages of sporocarp development. Condition specific nodes are highlighted

Figure S5 Differentially expressed genes in early, middle and late stage caps and stipes of *L. bicolor* carpohore. A Double hierarchical clustering of 6070 genes with differential expression in minimum two carpophore stages (fold change >log2 1, FDR p-value< 0.05). Eight cluster with distinct expression pattern are highlighted by coulours. Expression values are coloured by minimum and maximum expression per gene. **C**ap.lt.ave, late cap average gene expression; cap.el.ave, early cap average gene expression; cap.md.ave, medium cap average gene expression; sti.el.ave, early stipe average gene expression; sti.md.ave, medium stipe average gene expression; sti.lt.ave, late stipe average gene expression .**B** Table summarizing % of genes in each of the eight clusters coding for SSPs, Cazymes, Proteases, Lipases or with Cell cycle KOG annotation. C TopGO biological process enrichment analysis (Fisher p-value<0.05) for the eight clusters highlighted in **A**.

Figure S6 Comparison of ectomycorrhiza (EcM) and fruiting body (FB) up-regulated transcripts compared to free-living mycelium (FLM). (A) Venn diagramm of shared and EcM or FB-only up-regulated transcripts. (B) Functional classification of EcM and FB-shared and EcM and FB-only up-regulated transcripts. SSP small-secreted proteins. (C) Enriched biological process (BP) GO terms identified using TopGO for EcM and FB shared induced transcripts and EcM and FB-only induced transcripts.
