# Supplementary material for: A Transcriptomic Atlas of the Ectomycorrhizal Fungus Laccaria bicolor
Source: Microorganisms. 2021 Dec 17;9(12):2612. doi: 10.3390/microorganisms9122612 (PMC8708209; doi:10.3390/microorganisms9122612)
Supplement: Supplementary file 1 [file microorganisms-09-02612-s001.zip › microorganisms-1464646-supplementary/Figure S1_A.pdf]

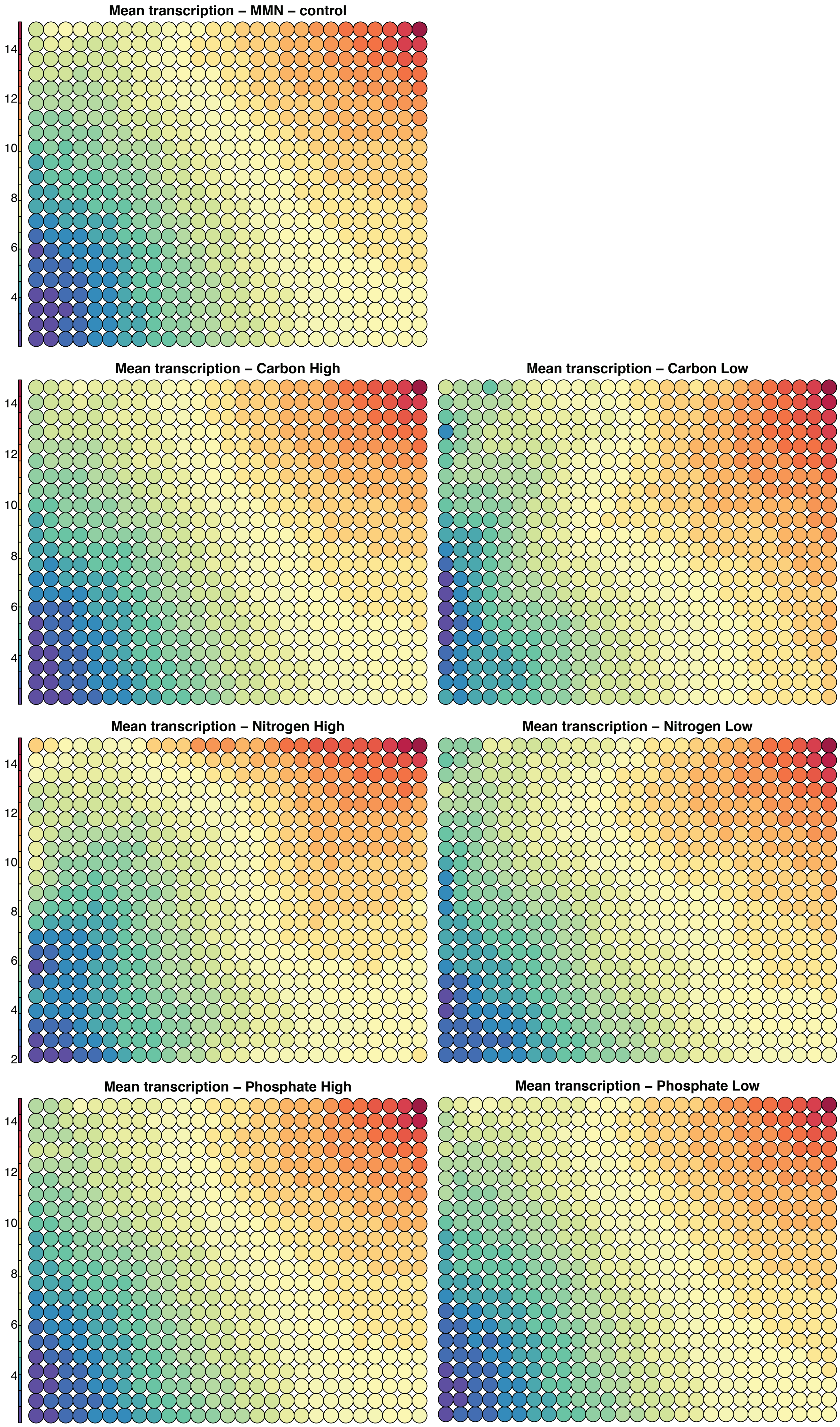
Mean transcription – MMN – control
14
12
10
8
6
4
Mean transcription – Carbon High
14
12
10
8
6
4
Mean transcription – Carbon Low
Mean transcription – Nitrogen High
14
12
10
8
6
4
2
Mean transcription – Nitrogen Low
Mean transcription – Phosphate High
14
12
10
8
6
4
Mean transcription – Phosphate Low
