# Supplementary material for: A Transcriptomic Atlas of the Ectomycorrhizal Fungus Laccaria bicolor
Source: Microorganisms. 2021 Dec 17;9(12):2612. doi: 10.3390/microorganisms9122612 (PMC8708209; doi:10.3390/microorganisms9122612)
Supplement: Supplementary file 1 [file microorganisms-09-02612-s001.zip › microorganisms-1464646-supplementary/Figure S5 C.pdf]

C

Cluster	TOP10 GO	TOP10 GO terms
Red	GO:0006355	regulation of transcription
	GO:0006096	glycolytic process
	GO:0009268	response to pH
	GO:0051090	regulation of DNA-binding transcription factor activity
	GO:0006526	arginine biosynthetic process
	GO:0006419	alanyl-tRNA aminoacylation
	GO:0006438	valyl-tRNA aminoacylation
	GO:0042351	de novo' GDP-L-fucose biosynthetic process
	GO:0009156	ribonucleoside monophosphate biosynthetic process
	GO:0006003	fructose 2,6-bisphosphate metabolic process
Green	GO:0006355	regulation of transcription
	GO:0007040	lysosome organization
	GO:0035023	regulation of Rho protein signal transduction
	GO:0008154	actin polymerization or depolymerization
	GO:0006665	sphingolipid metabolic process
	GO:0015904	tetracycline transport
	GO:0015947	methane metabolic process
	GO:0009439	cyanate metabolic process
	GO:0046677	response to antibiotic
	GO:0051056	regulation of small GTPase mediated signal transduction
Orange	GO:0009253	peptidoglycan catabolic process
	GO:0016998	cell wall macromolecule catabolic process
	GO:0006597	spermine biosynthetic process
	GO:0008295	spermidine biosynthetic process
	GO:0006097	glyoxylate cycle
	GO:0006437	tyrosyl-tRNA aminoacylation
	GO:0051341	regulation of oxidoreductase activity
Magenta	GO:0006730	one-carbon metabolic process
	GO:0005975	carbohydrate metabolic process
	GO:0006032	chitin catabolic process
	GO:0006468	protein phosphorylation
	GO:0006629	lipid metabolic process
	GO:0042742	defense response to bacterium
	GO:0050832	defense response to fungus
	GO:0007131	reciprocal meiotic recombination
	GO:0017183	peptidyl-diphthamide biosynthetic process
	GO:0008643	carbohydrate transport
Blue	GO:0006412	translation
	GO:0042254	ribosome biogenesis
	GO:0015986	ATP synthesis coupled proton transport
	GO:0006334	nucleosome assembly
	GO:0016071	mRNA metabolic process
	GO:0006457	protein folding
	GO:0045454	cell redox homeostasis
	GO:0006414	translational elongation
	GO:0006122	mitochondrial electron transport, ubiquitol to cytochrome c
	GO:0045900	negative regulation of translational elongation
Yellow	GO:0008152	metabolic process
	GO:0031119	tRNA pseudouridine synthesis
	GO:0008610	lipid biosynthetic process
	GO:0000272	polysaccharide catabolic process
	GO:0006807	nitrogen compound metabolic process
	GO:0006108	malate metabolic process
	GO:0043044	ATP-dependent chromatin remodeling
	GO:0031554	regulation of DNA-templated transcription, termination
	GO:0000290	deadenylation-dependent decapping of nuclear-transcribed mRNA
Turquoise	GO:0008643	carbohydrate transport
	GO:0006066	alcohol metabolic process
	GO:0019430	removal of superoxide radicals
	GO:0006562	proline catabolic process
	GO:0006014	D-ribose metabolic process
	GO:0006788	heme oxidation
	GO:0006725	cellular aromatic compound metabolic process
	GO:0006537	glutamate biosynthetic process
	GO:0016310	phosphorylation
	GO:0006810	transport
Light green	GO:0007172	signal complex assembly
	GO:0006298	mismatch repair
	GO:0006571	tyrosine biosynthetic process
	GO:0015931	nucleobase-containing compound transport
	GO:0009058	biosynthetic process
	GO:0006760	folic acid-containing compound metabolic process
	GO:0055070	copper ion homeostasis
	GO:0006559	L-phenylalanine catabolic process
