# Supplementary material for: A Transcriptomic Atlas of the Ectomycorrhizal Fungus Laccaria bicolor
Source: Microorganisms. 2021 Dec 17;9(12):2612. doi: 10.3390/microorganisms9122612 (PMC8708209; doi:10.3390/microorganisms9122612)
Supplement: Supplementary file 1 [file microorganisms-09-02612-s001.zip › microorganisms-1464646-supplementary/Figure S6.pdf]

A

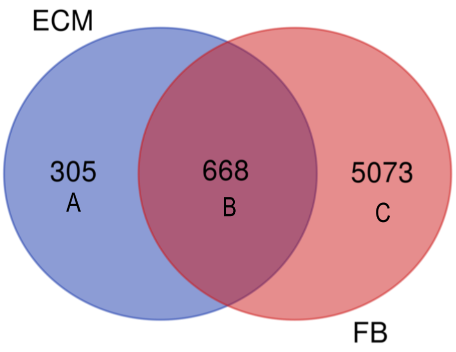

B

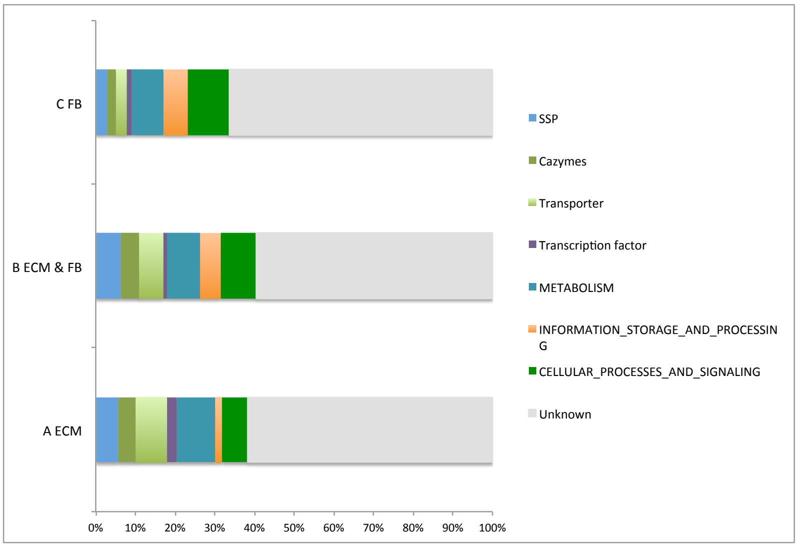

C

**GO.ID**	**GO**	**Term**	**A** ECM up	**B** ECM & FB up	**C** FB up
GO:0006066	BP	alcohol metabolic process		0.02136	
GO:0006865	BP	amino acid transport		0.0001	
GO:0015986	BP	ATP synthesis coupled proton transport			0.01479
GO:0019722	BP	calcium-mediated signaling		0.032	
GO:0005975	BP	carbohydrate metabolic process		0.0239	0.00393
GO:0008643	BP	carbohydrate transport	0.037		
GO:0016998	BP	cell wall macromolecule catabolic processes		0.014	
GO:0006952	BP	defense response	0.038		
GO:0008152	BP	metabolic process			0.02141
GO:0006120	BP	mitochondrial electron transport, NADH t...			0.00819
GO:0009116	BP	nucleoside metabolic process			0.00593
GO:0006334	BP	nucleosome assembly			0.00021
GO:0015780	BP	nucleotide-sugar transmembrane transport			0.03847
GO:0006730	BP	one-carbon metabolic process			0.00801
GO:0009253	BP	peptidoglycan catabolic process		0.003	
GO:0006813	BP	potassium ion transport		0.0095	
GO:0006470	BP	protein dephosphorylation		0.0087	
GO:0006885	BP	regulation of pH			0.00819
GO:0006465	BP	signal peptide processing			0.00059
GO:0008272	BP	sulfate transport		0.003	
GO:0006810	BP	transport	9.80E-10	0.0269	
