# Supplementary material for: A Transcriptomic Atlas of the Ectomycorrhizal Fungus Laccaria bicolor
Source: Microorganisms. 2021 Dec 17;9(12):2612. doi: 10.3390/microorganisms9122612 (PMC8708209; doi:10.3390/microorganisms9122612)
Supplement: Supplementary file 1 [file microorganisms-09-02612-s001.zip › microorganisms-1464646-supplementary/Supplementary_Information.pdf]

Supplementary Information

A transcriptomic atlas of the ectomycorrhizal fungus *Laccaria bicolor*

Joske Ruytinx, Shingo Miyauchi, Sebastian Hartmann-Wittulsky, Maíra de Freitas Pereira, Frédéric Guinet, Jean-Louis Churin, Carine Put, François Le Tacon, Claire Veneault-Fourrey, Francis Martin, and Annegret Kohler.

Supplementary note

1. Method overview for analysing transcriptomes
2. Evaluation of transcriptomes for free-living mycelium exposed to different nutritional conditions
3. Evaluation of transcriptomes for ECM development
4. Evaluation of transcriptomes for different stages of carpophore maturation
5. References

1. *Method overview for analyzing transcriptomes*

1.1. Preparation and evaluation of transcriptomic data

RNA-seq data were produced and processed as described in material and methods section of the main manuscript. Reads were mapped to the *Laccaria bicolor* genome (Lacbi2, JGI mycocosm) with CLC genomics workbench. Normalized read counts were produced with DESeq2 (Love *et al.*, 2014) and subsequently log2 transformed. Consistency of fungal transcriptomes was evaluated by examining genome-wide fungal transcripts from the RNA-seq data via three independent ways. (1) Density and distribution of normalized log2 reads from biological replicates was assessed for similarity to confirm appropriate growth and consistent sequencing. (2) Spearman's rank correlation was calculated with normalized read counts for all biological replicates. Similarity of transcriptomic profiles among biological replicates confirms consistency of the experiment. (3) Expression of housekeeping genes under all conditions was investigated. A stable, regular transcription level of selected housekeeping genes (i.e. chitin synthase, NADH dehydrogenase) suggests a normal physiological state of replicates in all conditions. After confirmation of consistency of biological data and similarity of biological replicates by these three independent evaluations, genes with an average read count exceeding 5 (per condition) were selected for the construction of transcriptomic models.

1.2. Construction of transcriptomic models for co-regulated gene analysis

Transcriptomic models were constructed using Self Organizing Maps (SOM) Harbouring Informative Nodes with Gene Ontology (SHIN+GO; Miyauchi *et al.*, 2016, 2017, 2018; 2020). A SOM was trained with the normalized read count of all replicates using Rsomoclu (Wittek *et al.*, 2013) on the computing cluster at INRAE Nancy to result in a clustering of genes with similar transcriptomic profile in nodes. The resolution of 25 genes per node was applied for clustering as empirically optimized by Miyauchi *et al*. (2017, 2018). The initial radius for SOM calculation was determined using a neighbour distance function in R kohonen (Wehrens and Buydens, 2007). The matrix of SOM nodes was visualized as a so-called Tatami map (Figure A). These Tatami maps are a series of dots, each representing a node containing genes with similar transcript profile. An average transcript profile for each node is calculated based on the transcript profile of individual gene members and is node specific. In Tatami maps, nodes are organized in a rectangular shape by which each node (dot) has four neighbouring nodes. Nodes are ordered according to their characteristic transcript profile, with neighbouring nodes showing more similarity to each other than nodes at a further distance in the rectangular. We examined condition specific and summary Tatami maps. In condition specific Tatami maps, nodes were color coded according to the average expression level in that particular condition. Nodes characterized by a contrasting expression level among experimental conditions change color when comparing color coded condition specific Tatami maps. Nodes showing a biased transcript level (up- or downregulated) at one (i.e. condition specific nodes) or few (shared responses) specific experimental condition were determined by a >2 log2 transcriptional difference between testing and control conditions. These nodes are indicated in a summary Tatami map and color coded according to the particular condition they contrast at. All procedures were performed with the SHIN module of the SHIN+GO platform.

1.3. Integration of functional annotations for L. bicolor genome

Functional annotation sets were combined using Carbohydrate Active Enzyme database (CAZy, Levasseur *et al*., 2013; Lombard *et al*., 2014), prediction of secretome (Pellegrin *et al*., 2015), InterPro (IPR; Finn *et al*., 2017), the Gene Ontology (GO; Blake *et al*., 2015), Kyoto Encyclopedia of Genes and Genomes (KEGG; Ogata *et al*., 1999) and Eukaryotic Orthologous Groups (KOG; Tatusov *et al*., 2003). The frequency of functional annotation terms per node of the transcriptomic model was calculated using tm package (Feinerer *et al*., 2008). The genes with the same annotation appearing more than twice in a single node were selected for the analysis. P values of enriched annotations per node were calculated using a function phyper in R stats package for the hypergenometric test (Johnson *et al*., 1997; R Core Team, 2015). P values were adjusted using Benjamini-Hochberg (FDR). Annotations with adjusted p values <0.01 were considered to be statistically significant. The nodes containing enriched functional terms were further selected with the condition-specificity determined with the SHIN module. All procedures were performed with the GO module.

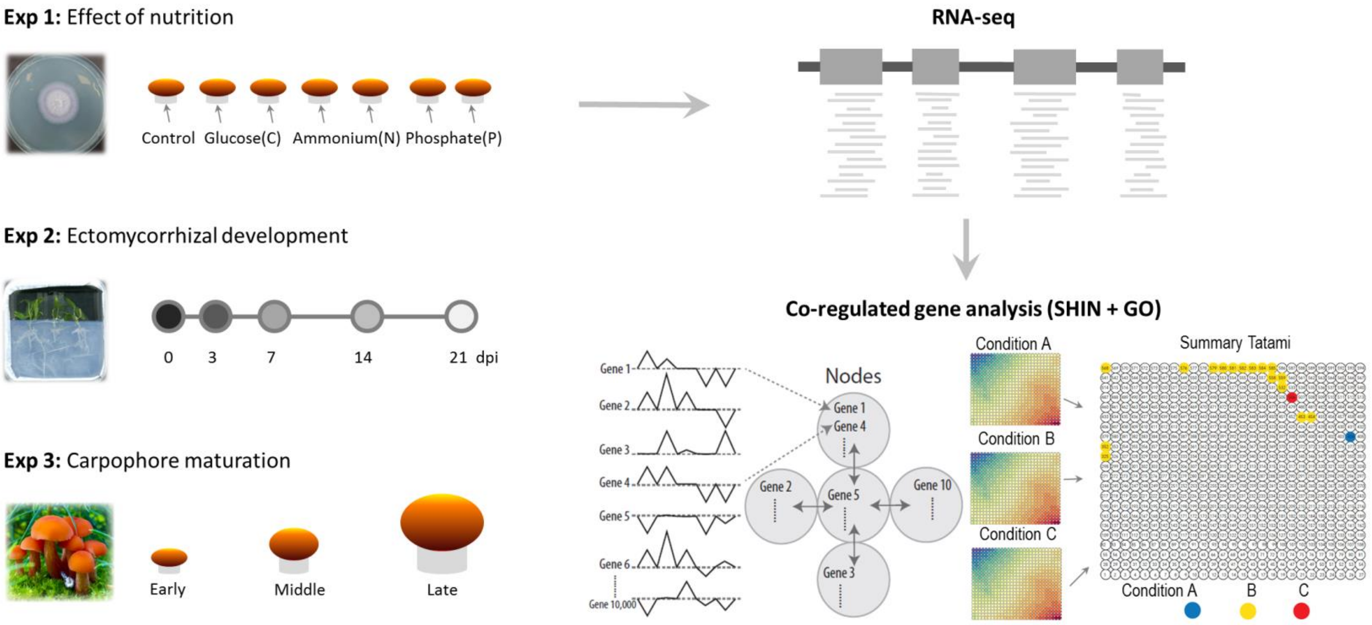

Figure A. Method overview for analysing transcriptomes. Total RNA was extracted from *L. bicolor* grown in the following experiments. Experiment 1: Free-living mycelium exposed to different nutritional conditions; Experiment 2: Ectomycorrhiza and extramatricular mycelium at different time points post inoculation of *P. tremula x alba*; Experiment 3: Carpophores of different ages. Transcripts were sequenced and quantified using RNA-seq. Co-regulated gene analysis was performed using SHIN+GO. An unsupervised machine learning method, Self-organizing maps (SOM) grouped genes showing similar transcriptional patterns into nodes. Nodes in close proximity contain genes with relatively similar transcriptional regulations. SOM of particular experimental condition is visualized as so-called Tatami maps. Transcriptomic Tatami maps exhibit nod-wise mean of the normalized transcription of genes per condition. Summary Tatami map represents nodes (i.e. gene groups) showing contrasting, highly differentially transcriptions in a particular condition.

2. *Evaluation of transcriptomes for free-living mycelium exposed to different nutritional conditions*

2.1 Evaluation of the normalised read counts of replicates

After the initial removal of low reads (< 5), 14,733 of 23,130 gene models were selected for further analysis. The density and distributions of normalised log2 reads from all biological replicates were examined (Fig. Ba). The box plot showed similar distributions of log2 read count with the median value of approximately 8.5 for all samples (Fig Ba left). There seemed to be no inconsistent replicates (outliers). Therefore, it was concluded that all 20 replicates were grown appropriately and their transcripts were consistently sequenced.

2.2 Correlation of individual biological replicates

A dendrogram was made based on the distances of transcriptomic similarity estimated with the replicates (Fig. Bb). Biological replicates grown under the identical conditions were similar to each other as the box formation of the replicates from the same conditions can be observed in the correlation matrix (Fig. Bb right). It indicated the biological replicates grew similarly under each of the conditions, confirming the consistency of the experiment. Globally, there seemed to be four major branches (Fig. Bb left). In two clusters at the top, the replicates grown with MMN media, high and low concentrations of phosphorus, and high concentration of carbon were closely clustered. This trend was also observed in the correlation matrix (Fig. Bb right). Meanwhile, the replicates from the low concentration nitrogen, low carbon, and high nitrogen were clustered independently (Fig. Bb right), suggesting that these three conditions induced somewhat dissimilar (or unique) transcriptomic responses.

2.3 Expression of housekeeping genes in all conditions

The transcription level of 33 housekeeping genes of 20 replicates from three growth stages was examined (Fig. Bc). Chitin synthase (12 genes) and NADH dehydrogenase (21 genes) were selected for the analysis. The expression of the housekeeping genes appeared to be more or less stable under the different conditions although there were some variations of the expression between the genes. The overall transcription patterns of the genes indicated that the physiological state of the replicates was normal and the transcription was consistent between all conditions. Thus, it was concluded that all 20 replicates were comparable at the transcriptomic level.

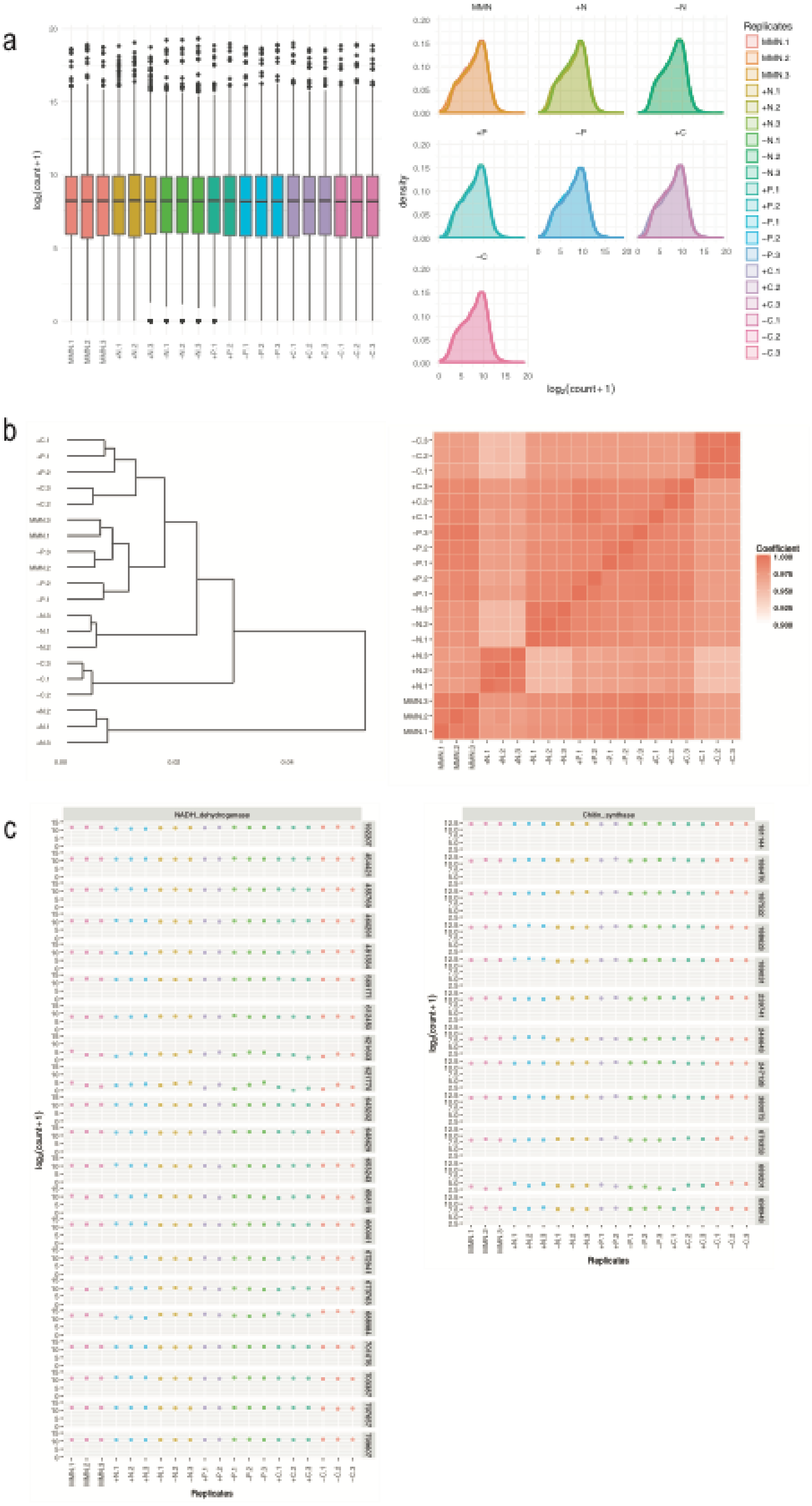

Figure B. Consistency of transcriptomic data for free-living mycelium with N, P, and C supply. a: Distribution and density of normalised log2 transformed read counts of genes from all biological replicates. **b:** Correlation of transcriptomes from the biological replicates. Left: Hierarchical clusters of biological replicates. Right: Correlation matrix of biological replicates **c:** Normalised log2 transformed read count of chitin synthase (12 genes) and NADH dehydrogenase (21 genes). **MMN**: MMN media. ±**N**: Nitrogen high or low concentrations. ±**P**: Phosphorus high or low concentrations. ±**C**: Carbon high or low concentrations.

3. <u>Evaluation of transcriptomes for ECM development</u>

3.1 Evaluation of the normalised read counts of replicates

After the initial removal of low reads (< 5), 15,341 of 23,130 genes were selected for further analysis. The density and distributions of normalised log2 reads from all biological replicates were examined (Fig. Ca). The box plot showed similar distributions of log2 read count with the median value of approximately 8.5 for all samples (Fig. Ca left). There seemed to be no inconsistent replicates (outliers). Therefore, it was concluded that all 37 replicates were grown appropriately and their transcripts were consistently sequenced.

3.2 Correlation of individual biological replicates

A dendrogram was made based on the distances of transcriptomic similarity estimated among all replicates (Fig. Cb). Biological replicates from the identical conditions were similar to each other as the box formation of the replicates from the same conditions can be observed in the correlation matrix (Fig. Cb right). It indicated the replicates grew similarly under each of the conditions, confirming the consistency of the experiment. Globally, there were two major branches (Fig. Cb left). The upper branch contained the replicates at the early time points (0, 3, 7 days) and the lower branch was made of the mid-late time points (14 and 21 days). The replicates at the early time points (0 and 3 days) were clustered on the top part of the tree.

Day 0 and 3 replicates had similar transcriptomic patterns, which include free-living (FLM), ectomycorrhizal (EcM), and extraradicular mycelia (ExM) at 3 days (FLM/EcM/ExM03) and FLM at 0 day (i.e. the background control for all replicates). The 7-day free-living mycelia were more similar to the replicates grown for 0 and 3 days. In contrast, EcM and ExM grown for 7 days were slightly distant from their replicates grown for 3 days. The observation suggests that some transcriptomic development occurred in EcM and ExM at 7 days compared to FLM at the same time point. The replicates grown for 14 and 21 days were grouped in the lower part of the tree (Fig. Cb left). FLM and ExM 14 and 21 days were grouped together while EcM 14 days were grouped with EcM 21 days, suggesting the transcriptomic responses of EcM became somewhat different those of FLM and ExM after 14 days. Please note that all 0 day-mycelia (the background control) are identical because they are made from 0 day-free living mycelia.

3.3 Expression of housekeeping genes in all conditions

The transcription level of 33 housekeeping genes of 37 replicates from three growth stages was examined (Fig. Cc). Chitin synthase (12 genes) and NADH dehydrogenase (21 genes) were selected for the analysis. Some genes coding for chitin synthase exhibited a gradual increase or decrease of the expression over time among all types of mycelia. For example, one gene (ProteinID 246643) showed a decrease at the point of 21 days while other genes (676353, 689201) had a progressive increase from 0 to 21 days. The expression of the housekeeping genes appeared to be more or less stable under the different conditions although there were some variations of the expression between the genes. The overall transcription patterns of the genes indicated that the physiological state of the replicates was normal and the transcription was consistent between all growth stages. Thus, all 37 replicates were comparable at the transcriptomic level.

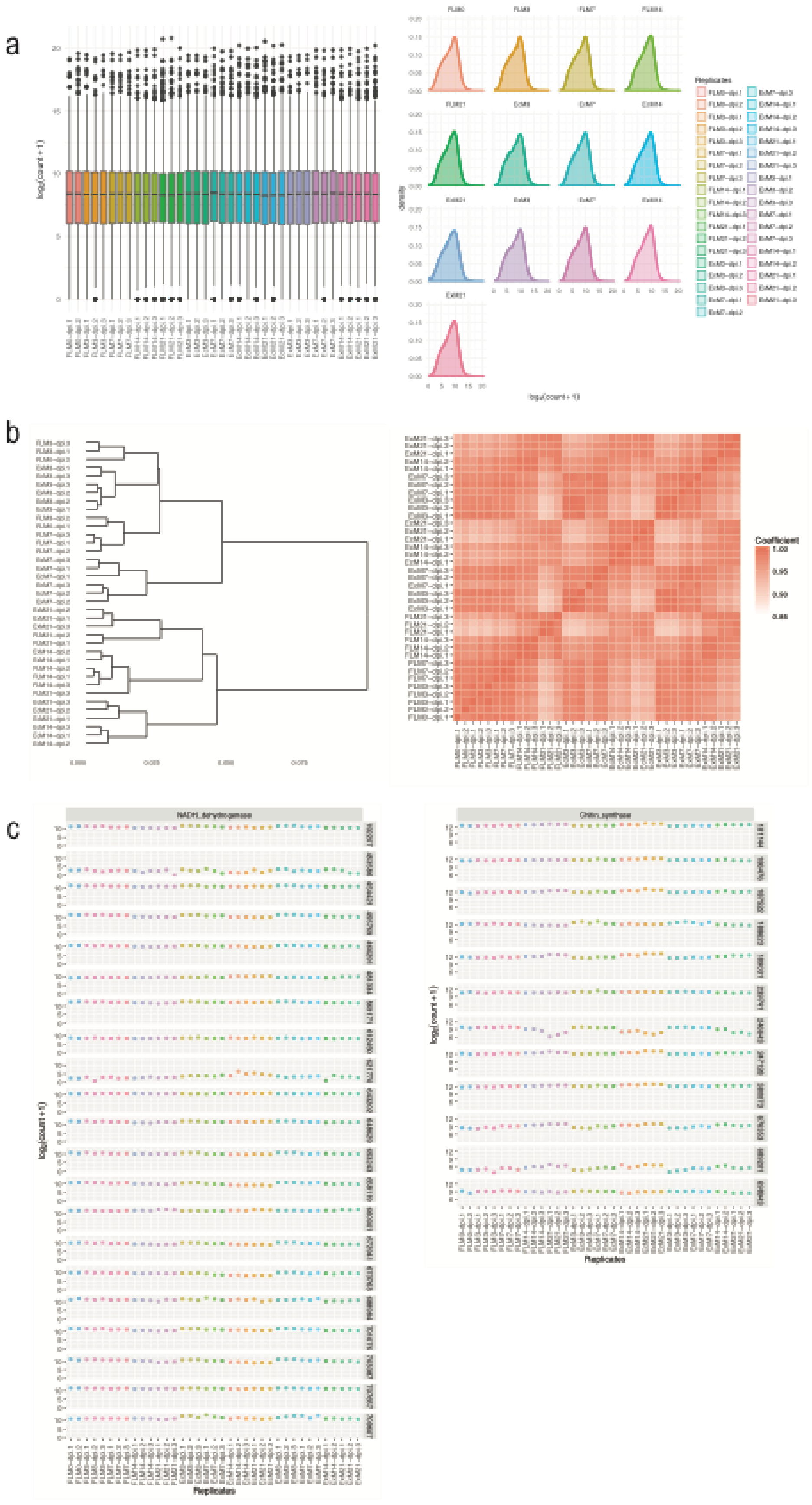

Figure C. Consistency of transcriptomic data for ECM development of *Laccaria bicolor* with *Populus tremula x alba*. a: Distribution and density of normalised log2 transformed read counts from all biological replicates. **b:** Correlation of transcriptomes from the biological replicates. Left: Hierarchical clusters of biological replicates. Right: Correlation marix of biological replicates **c:** Normalised log2 transformed read count of chitin synthase (12 genes) and NADH dehydrogenase (21 genes). **FLM/EcM/ExM**: Free-living, ectomycorrhizal, extraradicular mycelia**. 00/03/07/14/21**: Period of growth in days.

4. *Evaluation of transcriptomes for different stages of carpophore maturation*

4.1 Evaluation of the normalised read counts of replicates

After the initial removal of low reads (< 5), 16,609 of 23,130 genes were selected for further analysis. The density and distributions of normalised log2 reads from all biological replicates were examined (Fig. Da). The box plot showed similar distributions of log2 read count with the median value of approximately 10 for all samples (Fig. Da left). There seemed to be no inconsistent replicates (outliers). Therefore, it was concluded that all 18 replicates were grown appropriately and their transcripts were consistently sequenced.

4.2 Correlation of individual biological replicates

A tree was made based on the distances of transcriptomic similarity estimated among all replicates (Fig. Db left). Biological replicates from the identical conditions were clustered together. It indicates the replicates grew similarly under each of the conditions, which confirmed the consistency of the experiment. There are mainly three groups in the hierarchical clusters (Fig. Db left); (i) stipes at the early and middle stage; (ii) caps at all three stages; (iii) stipes at the late stage. The third group, stipes at the late stage, is the furthest in the tree, suggesting that their transcriptomic patterns were somewhat dissimilar to the other two groups. The correlation matrix showed the formation of two boxes for both caps and stipes (Fig. Db right), suggesting that the transcriptomic patters of cap/stipe tissues at the early and middle stages were more similar in comparison to those at the late stage. It suggests that the transcriptome of the early stage differs from that of the late stage. Overall, the obtained biological data were consistent and the biological replicates were comparable to each other.

4.3 Expression of housekeeping genes in all conditions

The transcription level of 38 housekeeping genes of 18 replicates from three growth stages was examined (Fig. Dc). Chitin synthase (12 genes) and NADH dehydrogenase (26 genes) were selected for the analysis. The expression of the housekeeping genes appeared to be more or less stable under the different conditions although there were some variations of the expression between the genes. It indicated that the physiological state of the replicates was normal and the transcription was consistent between all growth stages. Thus, all 18 replicates were comparable at the transcriptomic level.

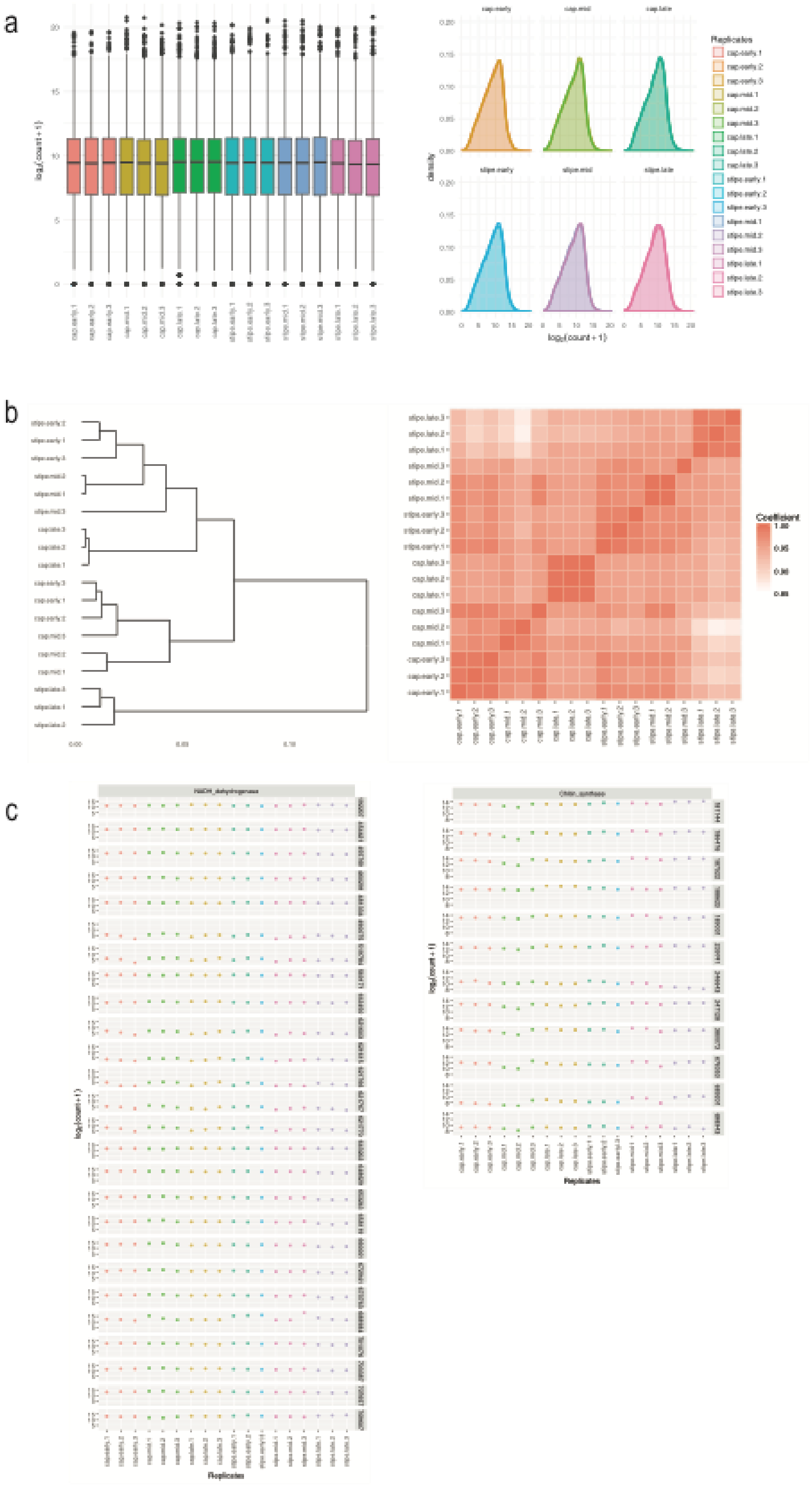

Figure D. Consistency of transcriptomic data for various growth stages of sporocarps. a: Distribution and density of normalised log2 transformed read counts of genes from all biological replicates. **b:** Correlation of transcriptomes from the biological replicates. Left: Hierarchical clusters of biological replicates. Right: Correlation matrix of biological replicates **c:** Normalised log2 transformed read count of chitin synthase (12 genes) and NADH dehydrogenase (26 genes). **Early/Mid/Late**: Three developmental stages of fungal growth.

5. References

1. Blake, J. A. *et al.* Gene ontology consortium: Going forward. *Nucleic Acids Res.* **43,** D1049–D1056 (2015).
2. Feinerer, I., Hornik, K. & Meyer, D. Text Mining Infrastructure in R. *J. Stat. Softw.* **25,** 1–54 (2008).
3. Finn, R. D. *et al.* InterPro in 2017—beyond protein family and domain annotations. *Nucleic Acids Res.* **45,** D190–D199 (2017).
4. Johnson, N. L., Kotz, S. & Balkrishnan, N. Discrete multivariate distributions John Wiley and Sons. *New York, New York, USA* 299 (1997).
5. Levasseur, A., Drula, E., Lombard, V., Coutinho, P. M. & Henrissat, B. Expansion of the enzymatic repertoire of the CAZy database to integrate auxiliary redox enzymes. *Biotechnol Biofuels*. **6**, 41 (2013).
6. Lombard, V., Golaconda Ramulu, H., Drula, E., Coutinho, P. M. & Henrissat, B. The carbohydrate-active enzymes database (CAZy) in 2013. *Nucleic Acids Res.* **42,** D490-5 (2014).
7. Miyauchi, S. *et al.* Visual Comparative Omics of Fungi for Plant Biomass Deconstruction. *Front. Microbiol.* **7,** 1335 (2016).
8. Miyauchi, S. *et al.* The integrative omics of white-rot fungus *Pycnoporus coccineus* reveals co-regulated CAZymes for orchestrated lignocellulose breakdown. *PLoS One* **1,** 1–17 (2017).
9. Miyauchi, S. et al. Integrative visual omics of the white-rot fungus *Polyporus brumalis* exposes the biotechnological potential of its oxidative enzymes for delignifying raw plant biomass. Biotechnol. Biofuels 11, 201 (2018).
10. Miyauchi, S. et al. Conserved white rot enzymatic mechanism for wood decay in the Basidiomycota genus Pycnoporus. DNA Res. 1–32 (2020). doi:10.1093/dnares/dsaa011
11. Ogata, H. *et al.* KEGG: Kyoto encyclopedia of genes and genomes. *Nucleic Acids Research* **27,** 29–34 (1999).
12. Pellegrin, C., Morin, E., Martin, F. M. & Veneault-Fourrey, C. Comparative Analysis of Secretomes from Ectomycorrhizal Fungi with an Emphasis on Small-Secreted Proteins. *Front. Microbiol.* **6,** 1278 (2015).
13. Wehrens, R. & Buydens, L. M. C. Self- and super-organizing maps in R: The kohonen package. *J. Stat. Softw.* **21,** 1–19 (2007).
14. Wittek, P., Gao, S. C., Lim, I. S. & Zhao, L. Somoclu: An Efficient Parallel Library for Self-Organizing Maps. (2013).
15. R Core Team. R: A language and environment for statistical computing. R Foundation for Statistical Computing. Vienna; 2013. http://www.r-project.org/
