# Supplementary figures and images for: A Transcriptomic Atlas of the Ectomycorrhizal Fungus Laccaria bicolor
Source: Microorganisms. 2021 Dec 17;9(12):2612. doi: 10.3390/microorganisms9122612 (PMC8708209; doi:10.3390/microorganisms9122612)

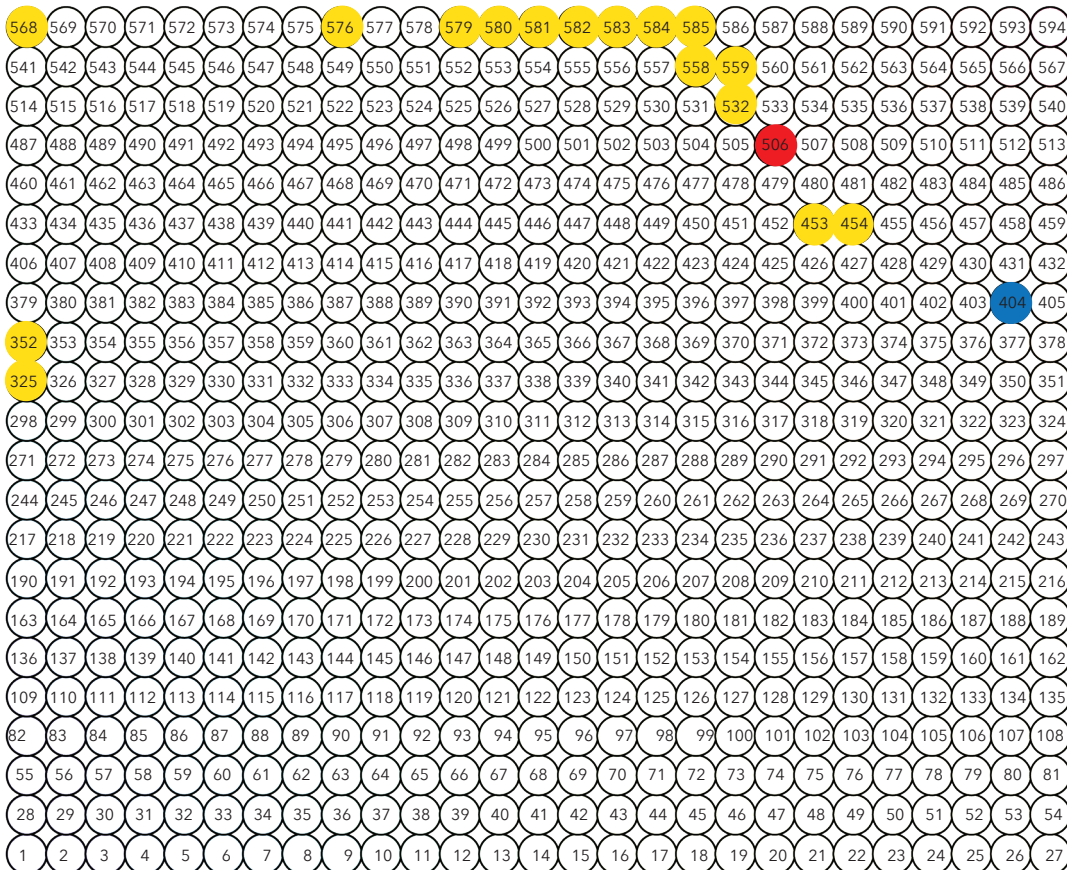

Carbon

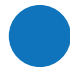

Nitrogen

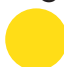

Phosphorus

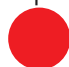

Supplement: Supplementary file 1 [file microorganisms-09-02612-s001.zip › microorganisms-1464646-supplementary/Figure S1_B.pdf]

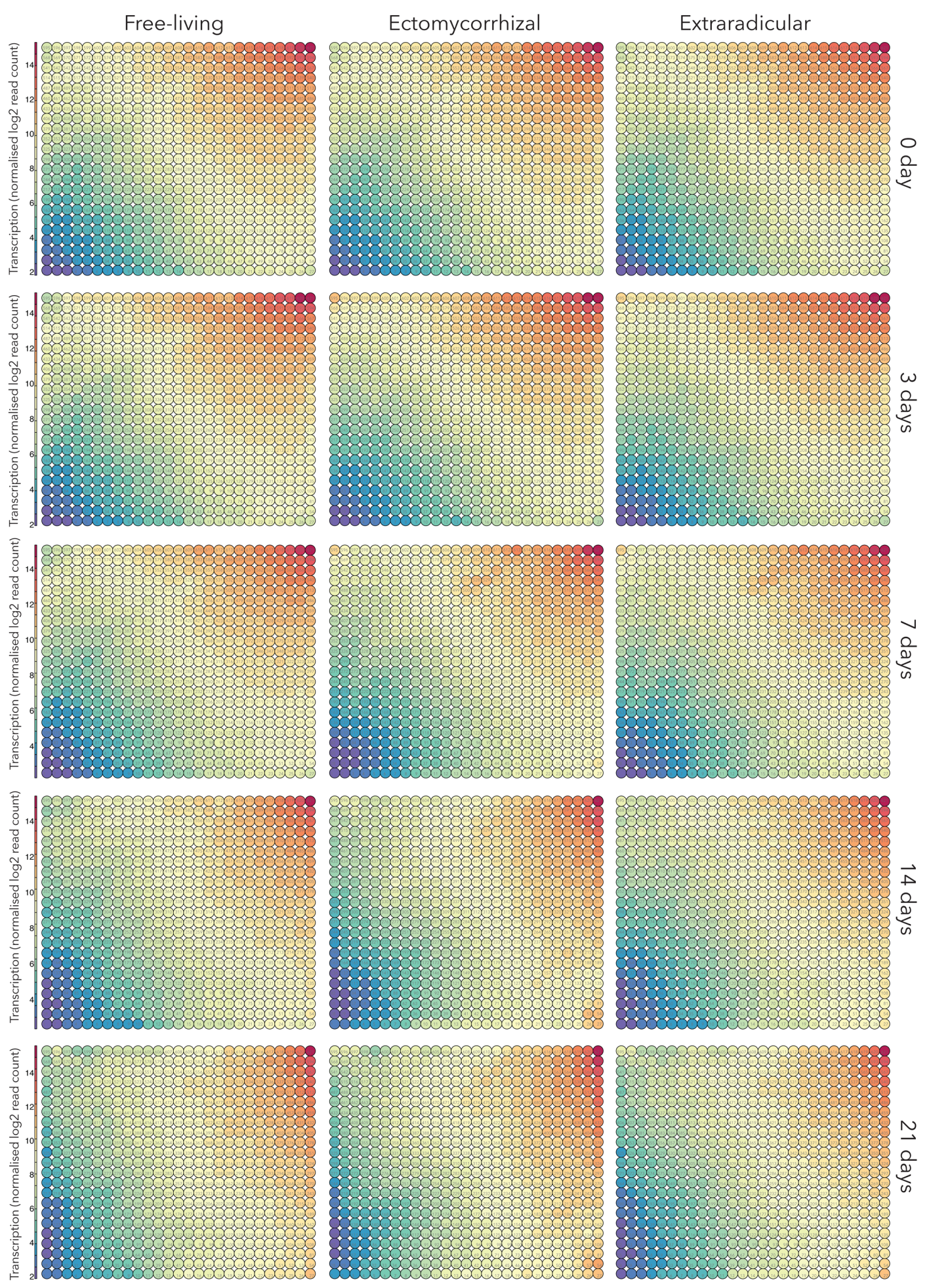

Supplement: Supplementary file 1 [file microorganisms-09-02612-s001.zip › microorganisms-1464646-supplementary/Figure S2_A.pdf]

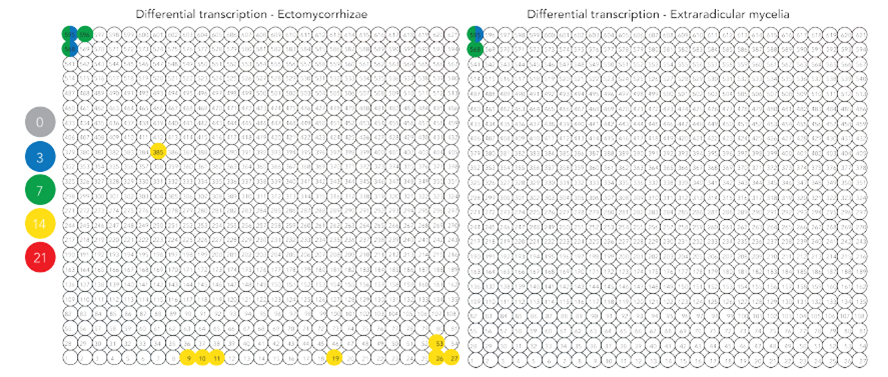

Supplement: Supplementary file 1 [file microorganisms-09-02612-s001.zip › microorganisms-1464646-supplementary/Figure S2_B.png]

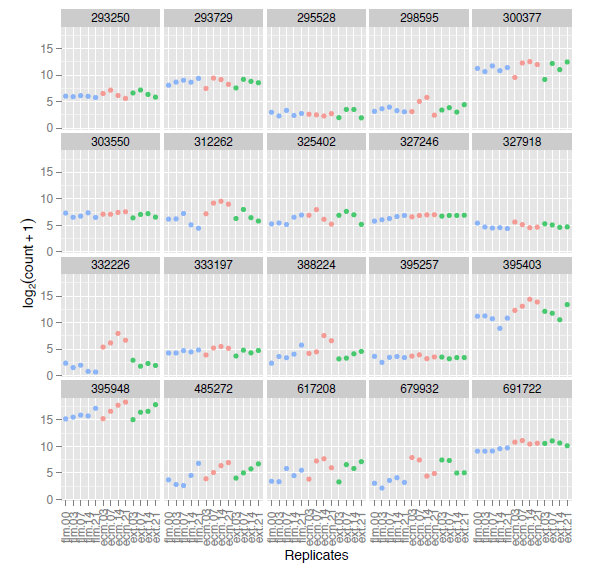

Supplement: Supplementary file 1 [file microorganisms-09-02612-s001.zip › microorganisms-1464646-supplementary/Figure S3_A.png]

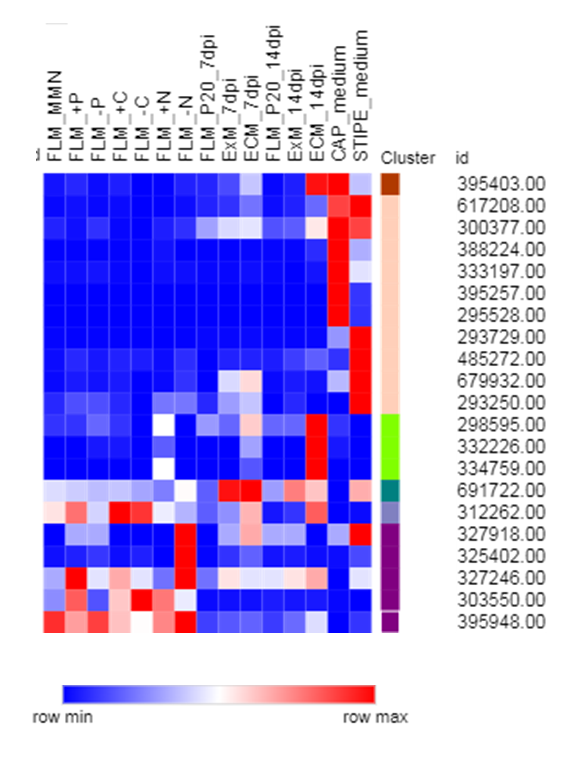

Supplement: Supplementary file 1 [file microorganisms-09-02612-s001.zip › microorganisms-1464646-supplementary/Figure S3_B.png]

## Caps

Early

Middle

Late

CvsS  
Middle

CvsS  
Late

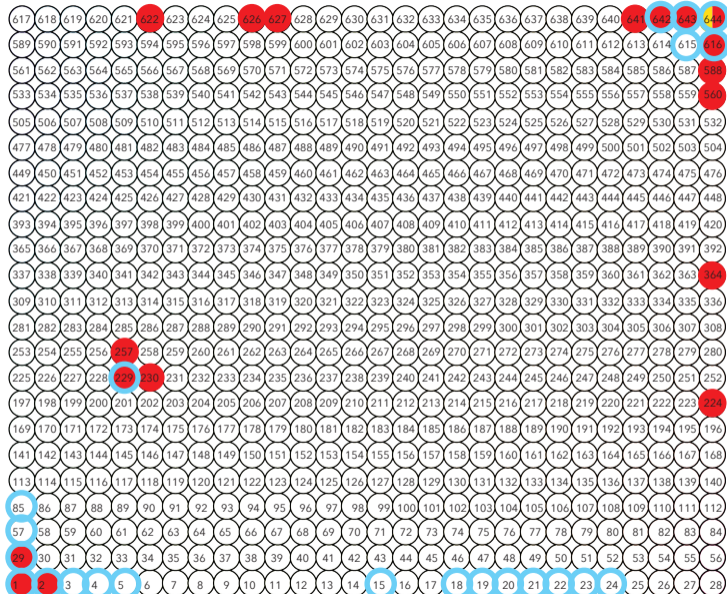

## Stipes

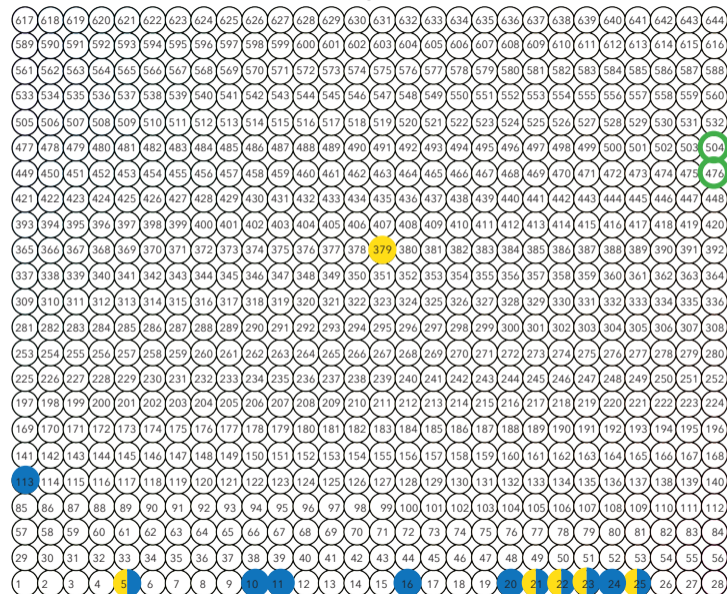

Supplement: Supplementary file 1 [file microorganisms-09-02612-s001.zip › microorganisms-1464646-supplementary/Figure S4.pdf]

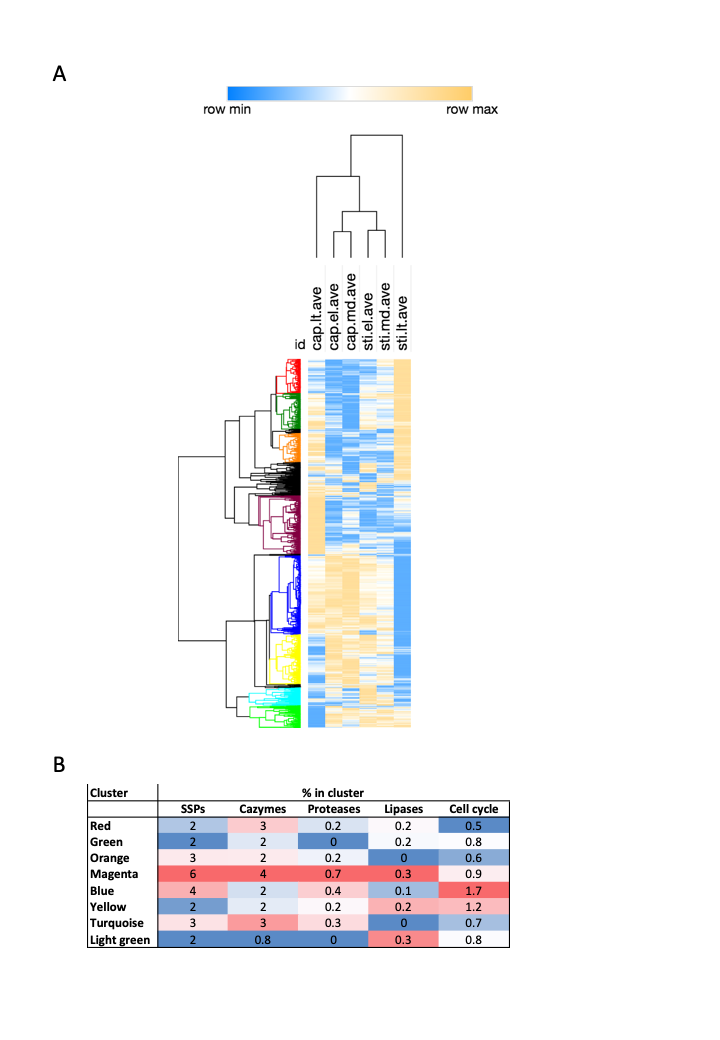

Supplement: Supplementary file 1 [file microorganisms-09-02612-s001.zip › microorganisms-1464646-supplementary/Figure S5 A_B.png]
